# Transdiagnostic Cognitive Control Training for Patients Waiting for Outpatient Psychotherapy: Randomized Clinical Trial

**DOI:** 10.2196/65867

**Published:** 2025-11-26

**Authors:** Maximilian Blomberg, Lisa Oberender, Ernst Koster, Timo Brockmeyer

**Affiliations:** 1 Department of Clinical Psychology and Translational Psychotherapy, Institute of Psychology, University of Münster, Fliednerstrasse 21, Münster, 48149, Germany, 49 2518334158; 2 Department of Clinical Psychology and Psychotherapy, Institute of Psychology, University of Göttingen, Göttingen, Germany; 3 Psychopathology and Affective Neuroscience Lab, Department of Experimental Clinical and Health Psychology, Ghent University, Ghent, Belgium

**Keywords:** cognitive control, emotion regulation, mHealth, eHealth, transdiagnostic approach

## Abstract

**Background:**

Various mental disorders are associated with impaired cognitive control, which is crucial for effective emotion regulation. Cognitive control training has demonstrated promise in enhancing emotion regulation and alleviating distress in disorders characterized by repetitive negative thinking, such as depression and anxiety.

**Objective:**

Given the importance of cognitive control and emotion regulation across mental disorders, this study investigates the efficacy of a mobile cognitive control training in a transdiagnostic outpatient sample awaiting psychotherapy.

**Methods:**

In this randomized clinical superiority trial with 2 parallel arms, 80 patients with various mental disorders from an outpatient waiting list received either 10 sessions of mobile cognitive control training using the Paced Auditory Serial Addition Test (PASAT) or an active control training using a speed of response task. The primary outcome was mental distress, measured by the Hopkins Symptom Checklist-11 (HSCL-11). Secondary outcomes included measures of cognitive control, rumination, repetitive negative thinking, difficulties in emotion regulation, cognitive emotion regulation, and disorder-specific symptoms. Outcomes were measured at baseline, post training, and at 3-month and 6-month follow-up.

**Results:**

Contrary to our primary hypothesis, cognitive control training was not superior in improving global mental distress directly after training (B=−.03, 95% CI –0.21, 0.16; *t*_179.60_=–0.26; *P*=.80; *d*=−0.04, 95% CI –0.35, 0.28); however, it led to greater improvements in cognitive control (B=−0.56, 95% CI –0.59,–0.54; *z*=−18.02; *P*<.001; *d*=−1.23, 95% CI −1.30,–1.20). This effect was similar at the 3-month and 6-month follow-up. Furthermore, at 3-month follow-up, cognitive control training resulted in fewer difficulties in emotion regulation (B=4.73, 95% CI 0.52, 9.12; *t*_177.99_=2.09; *P*=.04; *d*=0.34, 95% CI 0.04, 0.65), and anxiety symptoms (B=2.94, 95% CI 0.38, 5.82; *t*_66.51_=2.09; *P*=.04; *d*=0.70, 95% CI 0.09, 1.38), although the latter refers to a small subsample of patients with anxiety disorders. At 6-month follow-up, cognitive control training led to more adaptive cognitive emotion regulation (B=−5.18, 95% CI −9.74,–0.41; *t*_180.90_=−2.16; *P*=.03; *d*=−0.40, 95% CI −0.75,−0.03), and less social anxiety (B=2.00, 95% CI 0.14, 3.81; *t*_43.43_=2.08; *P*=.04; *d*=0.66, 95% CI 0.05, 1.24). The groups did not differ in any other outcome at any point in time.

**Conclusions:**

This study is the first to assess the efficacy of a mobile cognitive control training using the PASAT in a transdiagnostic outpatient sample. There was no evidence for the training’s efficacy on global mental distress and only weak evidence for the superiority in measures of emotion regulation and anxiety at follow-ups. Potential mediating pathways and moderating factors, such as the number of training sessions, should be investigated in larger studies.

## Introduction

### Background

Mental disorders are a major contributor to global disability and are associated with increased mortality, somatic comorbidities, and substantial socioeconomic costs [1,2]. While meta-analyses confirm the efficacy of psychotherapy and pharmacotherapy [3,4], effect sizes remain modest and relapse rates are high [5], underscoring the need for more effective treatments.

Frameworks such as the Research Domain Criteria (RDoC) propose a transdiagnostic approach to investigating shared risk factors among mental disorders [6,7]. Emotion regulation is one such factor implicated in the onset, maintenance, and relapse of various disorders [8-10]. Emotion regulation refers to the process by which individuals modulate the quality, intensity, and timing of their emotional responses [11]. Crucially, emotion regulation skills predict symptom improvement across disorders and treatments [12-17], and emotion regulation is targeted by many psychotherapeutic interventions [18-20].

Since Ochsner and Gross’s foundational work [21,22], research has increasingly focused on to what extent emotion regulation depends on cognitive control [23-25]. Cognitive control refers to a set of interrelated processes, largely mediated by the prefrontal cortex, that support goal-directed behavior [26,27]. These processes include shifting (flexibly switching between tasks or mental sets), inhibition (suppressing automatic or prepotent responses), and updating (maintaining and renewing relevant information in the working memory while discarding irrelevant material) [28]. A multitude of meta-analyses have shown that numerous mental disorders are associated with reduced cognitive control, covering all domains of shifting, inhibition, and updating (see [29] for a review of meta-analyses).

Notably, impairments in cognitive control, especially difficulties with inhibition and working memory updating, have been associated with maladaptive emotion regulation strategies [23,30-34]. As outlined in the impaired disengagement hypothesis [35], rumination serves as an illustrative case of how cognitive control impairments contribute to maladaptive emotion regulation. According to this hypothesis, individuals with impaired cognitive control struggle to divert their attention and focus from negative (self-referential) information in working memory. This prolongs a ruminative inward focus on the negative material that increases negative affect and thus lowers the threshold for additional negative information to enter working memory [35]. This cycle of increased rumination, qualified by impaired cognitive control, has been supported by meta-analytic findings on the links between rumination and difficulties in discarding no longer relevant material from working memory [36].

Given the links between impaired cognitive control and maladaptive emotion regulation, interventions that enhance cognitive control may offer a promising avenue for alleviating mental distress rooted in such dysfunctional emotion regulation. One commonly used approach to improve cognitive control is based on training using the Paced Auditory Serial Addition Test (PASAT) [37,38]. In the PASAT, participants hear a continuous stream of single-digit numbers ranging from 1 to 9. They are instructed to continuously add and respond to the sum of the last 2 numbers they heard. For example, if they last heard the numbers 2 and 6, the correct answer is 8. If they hear the number 9 next, the correct answer then is 15 (because 6+9=15). This requires them to constantly update their working memory by replacing the next-to-last heard number with the most recently heard number to compute the updated sum. Furthermore, the PASAT requires inhibition, since participants have to suppress responses to the no longer relevant numbers and have to inhibit immediate responses to the current number (before summing the numbers up). As such, the PASAT engages cognitive control processes such as working memory updating and inhibition [28].

PASAT-based cognitive control training has shown efficacy in reducing symptoms of depression and maladaptive emotion regulation, such as rumination, in individuals with current depressive episodes [39-43], those at elevated risk [44], and patients in remission [45-47]. In these intervention studies, the PASAT has frequently been compared to an active control condition that focused on speed of responses (ie, respond immediately to the last heard number) and thus does not target core cognitive control processes such as updating of working memory [48]. However, the benefits of the training have not been observed in nonclinical samples of healthy students [49-51], older adults [52], or high trait ruminators [53]. Moreover, PASAT-based training has not shown an additive benefit when combined with behavioral activation [54] or when used as an adjunct to inpatient treatment [55]. Thus, while promising for clinical populations, its effectiveness appears limited in nonclinical samples and may be limited when combined with other treatments.

### Objectives

Most intervention studies using the PASAT have focused primarily on individuals with depression or an increased vulnerability for depression. This may have overlooked the broader applicability of cognitive control training across the spectrum of mental disorders. Given the well-documented impairments in cognitive control and maladaptive emotion regulation across various mental disorders [8,29], this study investigates the efficacy of PASAT-based cognitive control training as a transdiagnostic intervention in a heterogeneous clinical sample waiting for psychotherapy in an outpatient clinic. Our primary hypothesis is that participants receiving PASAT-based cognitive control training will show a significantly greater reduction in mental distress from baseline to post training (primary end point). This effect is expected to exceed that of an active control condition, which emphasizes response speed but does not target cognitive control processes. Based on previous evidence for the intervention’s efficacy in reducing rumination in individuals with depression [48], we further hypothesize that cognitive control training will lead to greater improvements in emotion regulation compared to the control condition. However, as a narrow focus on rumination may miss broader emotion regulation impairments across diagnoses [56], we will assess this outcome using multiple measures, including a repetitive negative thinking style, general difficulties in emotion regulation, and cognitive emotion regulation strategies. To explore the broader functional significance of training-induced improvements, we will also examine effects on work and social adjustment and quality of life. Finally, to capture potential improvements at the symptom level, we additionally examine changes in disorder-specific symptoms. To assess potential longer-term effects of the training, follow-up assessments are conducted not only post training but also at 3 and 6 months.

## Methods

### Design

Participants were randomly assigned (1:1 ratio) to receive either a cognitive control training or an active control training (ie, a training that focused on response speed). Randomization (without stratification) was carried out automatically with the *psych* package, version 2.2.9 [57] directly before the first training session. We used the online survey framework formr [58] throughout the entire study to conduct the assessments and deliver the interventions. The computerized allocation to training conditions was concealed from the study personnel. Only after a phone call at the time when half of the training sessions were completed (intended to enhance engagement following the first 5 training sessions, see section “Treatments”) could participants reveal their condition to the study personnel. Importantly, the study personnel never inquired about details of the participants’ training procedures that could disclose their conditions. Upon recruitment, participants were blind to the nature of the different training conditions. They were informed that during the training, they would engage in a task that demands their concentration. After randomization, they only received specific information about their respective condition. All instructions were written and standardized. All outcomes were either self-report measures or computerized tasks, so that no masking of assessors was required. Assessments of baseline, post training, 3-month, and 6-month follow-up data of all self-report questionnaires were conducted independently by the patients through formr. Training and assessment tasks were programmed using JsPsych [59] and JATOS [60]. This trial was conducted in line with the CONSORT (Consolidated Standards of Reporting Trials) guidelines (see Checklist 1).

### Participants

Between May 2021 and November 2023, a total of 80 patients were recruited from the waiting list of the outpatient psychotherapy clinic at the Institute of Psychology of the University of Göttingen, Germany, which offers cognitive behavioral therapy. Forty patients (50%) were randomly assigned to the cognitive control training and 40 patients to the speed of response training (active control). Patients were initially made aware of the study with the help of leaflets upon registering for the waiting list of the treatment facility. Patients were eligible for the study if they were between 18 and 65 years old and met the diagnostic criteria for one or more of the following mental disorders that had been associated with cognitive control impairments [29]: major depressive disorder (including recurrent or persistent depressive disorder), agoraphobia, panic disorder, social anxiety disorder (SAD), specific phobia, generalized anxiety disorder, obsessive-compulsive disorder, posttraumatic stress disorder, somatic symptom disorder, eating disorder (anorexia nervosa, bulimia nervosa, and binge-eating disorder), substance use disorder (abstinent for at least 3 months), borderline personality disorder, or adjustment disorder. To maximize external validity, exclusion criteria were kept at a minimum: nonabstinent substance use disorder, severe psychiatric comorbidity (eg, schizophrenia), medical instability, dementia, acute suicidality, or insufficient knowledge of the German language to understand the questionnaires and instructions. Diagnostic eligibility was assessed according to the *DSM-5* (*Diagnostic and Statistical Manual of Mental Disorders* [Fifth Edition]) [61], using the *Diagnostic Interview for Mental Disorders* [62]. Screening of adjustment disorder was conducted using the relevant section from the *Structured Clinical Interview for DSM-5 Disorders* [63], since this diagnosis is not covered by the *Diagnostic Interview for Mental Disorders*. Interviews were conducted by clinical psychology graduate students with master’s degrees, trained and supervised by a licensed psychotherapist with extensive experience in both interview formats.

Sample size was determined by an a priori power analysis using G*Power [64]. Based on previous findings in a similar study [45], this study was powered to detect a moderate within-between (time x training group) interaction effect of *f*=0.25 in a repeated-measures design for the primary hypothesis that the cognitive control training would reduce global mental distress from baseline to posttraining assessment significantly more than the speed of response training. With *α*=.05, statistical power 1–β=.95, and while accounting for an approximate 25% of data loss, a minimum sample size of 72 would have been sufficient, which was conservatively rounded up to 80, following recommendations for pilot trials [65].

### Treatments

#### Overview

Participants performed 10 online training sessions of either the cognitive control training or the speed of response training over the course of 2 weeks. They conducted these sessions independently using their smartphones via a web browser, enabling a flexible and remote completion of the intervention. According to meta-analytic findings, conducting cognitive control training remotely instead of in a laboratory setting does not negatively affect its efficacy [66]. Additionally, remote training provides a more ecologically valid context. Immediately before the first training session, both groups watched a standardized psychoeducative video that was specifically created for this study and that aimed to increase task engagement [41]. For both conditions, the video highlighted that participating in a cognitive training with a demanding task necessitates sustained attention and focus even if frustrating negative thoughts may emerge. The video and its transcript can be found on the project’s Open Science Framework page [67]. After completing the first 5 training sessions, participants received a short phone call from the study personnel with the purpose of motivating participants to complete the remaining 5 sessions. During the call, participants’ completion of the first half was positively acknowledged, and they were encouraged to finish the second half. The call also included a discussion on overcoming potential obstacles and improving conditions for training completion. For example, advice was given on training in a private, quiet environment and using headphones to minimize any distraction from the task.

#### Cognitive Control Training

Participants in the cognitive control training group underwent 10 training sessions, each lasting 20 minutes, using an adaptive version of the PASAT (aPASAT) that has been used in cognitive control training studies before [45,49]. During training, participants hear a continuous stream of single digits (1-9) and must calculate and respond with the sum of the 2 most recently presented digits, selecting the correct answer from options on the screen ranging from 1 to 18. To tailor the training to the individual’s performance level, the speed of digit presentation was adjusted dynamically. The interstimulus interval (ISI) between 2 digits began at 3000 milliseconds for each training session and was decreased or increased by 100 milliseconds after every sequence of 4 consecutive correct or incorrect responses, respectively. Besides an initial set of 8 practice trials to familiarize participants with the task, each training session consisted of 400 trials. Consistent with prior research, training progress was monitored using the median ISI levels achieved in each session [45,49].

#### Speed of Response Training

Participants in the speed of response training group received the same number of training sessions and trials as participants in the cognitive control training group. Contrary to the cognitive control training, patients receiving the control training based on the Speed of Response Task (SOR) [49], in which participants just respond as fast as possible to the last heard number. Therefore, this training does not target the same cognitive processes as the adaptive PASAT but captures response speed and sustained attention. The same criteria for the starting ISI and changes according to performance are applied.

### Outcomes

#### Timeline for Assessments

All outcomes were assessed at baseline, directly following the training, and then again at 3-month and 6-month follow-up. In addition to assessing immediate training effects, we selected these follow-up time points commonly used in cognitive control training studies, as they may capture longer-term effects that only emerge over time [48]. Unlike the other measures, quality of life and work and social adjustment were only assessed at baseline and at 3-month and 6-month follow-up to reduce the time burden for participants and because training was not expected to lead to meaningful changes in these outcomes within such a short period of time.

#### Primary End Point: Global Mental Distress

The level of global mental symptom distress was assessed using the Hopkins Symptom Checklist-11 (HSCL-11) [68], a self-administered questionnaire with robust psychometric characteristics that measures a range of mental symptoms, such as anxiety and depression. Each of the 11 items is rated on a Likert scale from 1 to 4 with higher scores indicating greater symptom distress (mean scores for the total scale thus range between 1 and 4). At baseline assessment, Cronbach α for the entire sample was 0.84. Given the transdiagnostic approach of the trial, the HSCL-11 was selected for its ability to assess mental distress that is not confined to the specific symptom pattern of a particular mental disorder but instead measures symptoms that are common across various mental disorders [68].

#### Secondary End Points

##### Rumination

Rumination was assessed using the 10-item version of the Response Style Questionnaire (RSQ-10) [69]. The RSQ-10 is a self-report questionnaire that measures rumination on a 4-point Likert scale from 1 to 4, with higher scores indicating a stronger tendency to ruminate (total score range: 10‐40). Reliability and validity of the German version have been demonstrated (RSQ-10D) [70]. Importantly, the brooding and reflection subscales of the RSQ-10 measure rumination above and beyond what can be explained by symptoms of depression [69]. At baseline, Cronbach α was 0.73. Following suggestions by the authors of the RSQ-10, we also conducted exploratory subanalyses focusing on the 5-item brooding subscale of the questionnaire, since brooding depicts the more dysfunctional component of rumination that is associated with mental disorders [69,71].

##### Repetitive Negative Thinking

Given the transdiagnostic nature of repetitive negative thinking and difficulties with disengagement of (content- and disorder-independent) negative thoughts [72], repetitive negative thinking was measured alongside rumination to assess this specific style of thinking. We used the Perseverative Thinking Questionnaire (PTQ) [73]. Each of the 15 items of the PTQ (eg, The same thoughts keep going through my mind again and again.) is scored on a Likert scale from 0 to 4 with higher scores displaying more repetitive negative thinking (total score range: 0‐60). At baseline, Cronbach α was 0.93.

##### Difficulties in Emotion Regulation

Difficulties in emotion regulation were assessed using the 16-item version of the Difficulties in Emotion Regulation Scale (DERS-16) [74]. The DERS-16 is a short form of the original 36-item DERS [75] which retains the good reliability and validity of the original scale. Difficulties in emotion regulation are assessed on a Likert scale from 1 to 5 with higher scores indicating greater difficulties in regulating emotions (total score range: 16‐80). At baseline, Cronbach α was 0.91.

##### Cognitive Emotion Regulation

Due to the expected specific nature of the challenges in emotion regulation linked to impairments in cognitive control, we complemented the evaluation of general difficulties in emotion regulation with a more targeted assessment of habitual cognitive emotion regulation using the Cognitive Emotion Regulation Questionnaire (CERQ) [76]. With 36 items which are scored on a Likert scale from 1 to 5, the CERQ measures cognitive emotion regulation on 9 subscales. Consistent with an earlier study on cognitive control training [45], we calculated compound scores for adaptive cognitive emotion regulation using items from the subscales “positive refocusing,” “planning,” “positive reappraisal,” “putting into perspective,” and “acceptance” (total score range: 20‐100) and for maladaptive cognitive emotion regulation using the items from the subscales “self-blame,” “other-blame,” “rumination,” “catastrophizing” (total score range: 16‐80). A German translation has been psychometrically validated [77]. At baseline, Cronbach α was 0.78 and 0.70 for the adaptive and maladaptive cognitive emotion regulation compound score, respectively.

##### Cognitive Control

Cognitive control was measured using a nonadaptive assessment version of the PASAT [37] during which participants performed 3 blocks of increasing difficulty (ISI block 1=3000 ms; ISI block 2=2000 ms; ISI block 3=1500 ms). The assessment consisted of a total of 180 trials, with 60 trials per block. Additionally, participants performed 8 practice trials beforehand. As a performance indicator, we analyzed trial data and fitted a model using a binomial error distribution on the probability of a correct response for an individual trial (refer to the section “Statistical Analysis”). This can also be interpreted as the expected ratio of correct trials per assessment (minimum 0, maximum 1).

##### Quality of Life

Quality of life was assessed using the World Health Organization Quality of Life Brief Form (WHOQOL-BREF) [78]. The WHOQOL-BREF is a transdiagnostic self-report measure to assess the health condition and impact of illnesses on daily activities and behavior in 4 domains (physical, psychological, social, and environmental) on a Likert scale from 1 to 5. Scores for each domain are converted to a range between 0 (worst) and 100 (best quality of life). Psychometric quality and culture-sensitive validity have been shown for the WHOQOL-BREF in several studies (eg, [78]). Cronbach α at baseline for the 4 domains ranged between 0.70 (social) and 0.83 (psychological).

##### Work and Social Adjustment

The Work and Social Adjustment Scale (WSAS) [79] is a 5-item self-report measure of functional impairment in work-related and social domains attributable to an identified problem. Items are rated on a Likert scale from 0 (not at all impaired) to 8 (very severely impaired). Total scores range between 0 and 40. Psychometric quality and validity of the WSAS for various disorders have been reported [79]. At baseline, Cronbach α was 0.74.

### Disorder-Specific End Points

Depending on their diagnoses, participants completed additional disorder-specific self-report questionnaires at baseline, post training, and at the 3- and 6-month follow-ups. Patients who met *DSM-5* criteria for major depressive disorder completed the Patient Health Questionnaire-9 (PHQ-9) [80]. Anxiety symptoms in patients with an anxiety disorder (excluding SAD) were assessed using the Generalized Anxiety Disorder Questionnaire (GAD-7) [81]. Participants diagnosed with SAD completed the Mini-Social Phobia Inventory (mini-SPIN) [82]. Other diagnoses were less common in our sample (each <5, see Table 1). Further details on disorder-specific outcome measures are provided in Multimedia Appendix 1.

**Table 1.** Sample characteristics.^a^

Variable	aPASAT^b^ training group (n=40)	SOR^c^ training group (n=40)	Total sample (N=80)
Age (years), mean (SD)	26.1 (6.3)	25.6 (3.9)	25.8 (5.2)
Women, n (%)	23 (57)	22 (55)	45 (56)
Psychotropic medication, n (%)	11 (28)	11 (28)	22 (28)
Education (years), mean (SD)	17.3 (3.4)	17.6 (3.3)	17.5 (3.3)
Diagnosis according to the *DSM-5^d^*, n (%)			
Major depressive disorder	31 (78)	34 (85)	65 (81)
Anxiety disorder	15 (38)	16 (40)	31 (39)
Posttraumatic stress disorder	2 (5)	1 (2)	3 (4)
Somatic symptom disorder	0 (0)	3 (8)	3 (4)
Social anxiety disorder	10 (25)	11 (28)	21 (26)
Borderline personality disorder	2 (5)	2 (5)	4 (5)
Eating disorder	0 (0)	0 (0)	0 (0)
Obsessive-compulsive disorder	2 (5)	0 (0)	2 (2)
Substance abuse disorder	0 (0)	0 (0)	0 (0)
Adjustment disorder	0 (0)	0 (0)	0 (0)

a Data are mean (SD) or n (%); anxiety disorder encompasses panic disorder, agoraphobia, specific phobia, and generalized anxiety disorder; eating disorder encompasses anorexia nervosa, bulimia nervosa, and binge eating disorder.

b aPASAT: adaptive Paced Auditory Serial Addition Test.

c SOR: Speed of Response Task.

d *DSM-5*: *Diagnostic and Statistical Manual of Mental Disorders *(Fifth Edition).

### Additional Measures

We measured treatment credibility and expectancy using the Credibility Expectancy Questionnaire (CEQ) [83] at baseline and post training. Additionally, we assessed the intake of psychotropic medication and monitored the commencement of psychotherapy between baseline and 6-month follow-up.

### Procedure

Participants were instructed to complete all study assessments and training on their smartphones. After screening for eligibility, patients returned home and completed the baseline assessment on the next day. They were instructed to complete 10 online training sessions within a 2-week period. Patients could only complete 1 training session per day. To increase adherence, depending on their preferences, participants received automated daily reminders either via email or SMS. We monitored training adherence by regularly checking the completion of training sessions remotely and phoned participants in case of strong deviations from the training schedule (ie, multiple days without completing a training session). Participants returned to the laboratory for the posttraining assessment, where they conducted all self-report questionnaires and the PASAT assessment on their smartphones. At this time, they also received instructions for the upcoming follow-up assessments and engaged in a short qualitative interview on their experience with the training. The results of these qualitative interviews will be reported elsewhere since they are beyond the scope of this quantitative assessment of the cognitive control training. Follow-ups were conducted remotely, starting after the final training session. Patients were debriefed after the last follow-up.

### Statistical Analysis

Primary analyses followed an intention-to-treat approach. Outliers (|z|>3) across outcomes were adjusted using winsorization to reduce the influence of extreme values on the analysis. However, results were cross-validated with unadjusted data to ensure robustness. All analyses were conducted using linear mixed-effects models [84] with the *lme4 package* version 1.1‐31 [85] in R (version 4.5.0; R Core Team) [86].

We compared the average outcome score trajectories between the 2 training conditions using random intercept models and fixed effects for time of assessment, condition, and their interaction. Time of assessment was modeled as a repeated-measures factor with 4 levels (baseline, post training, 3-month, and 6-month follow-up), while training condition was a between-subjects factor (aPASAT vs SOR). For quality-of-life outcomes, time had 3 levels: baseline, 3-month follow-up, and 6-month follow-up. We fitted a model using a binomial error distribution to analyze the data from the PASAT assessment version to measure cognitive control, since PASAT performance is bound between 0 and 1 (portion of correct trials) and therefore not normally distributed. This yields a z-statistic. As performance is expected to change given different ISI, we included the current ISI as a covariate.

As a first step, we compared a full model including time of assessment, training condition, and their interaction to a reduced model with time of assessment as the sole predictor. This approach guards against “cryptic multiple testing,” that is, inflated false positives from interpreting multiple time point–specific effects without first establishing a significant overall interaction [87]. We then examined specific interaction effects between training condition and time of assessment for each interval: from baseline to post training, and to the 3- and 6-month follow-up (3 group-by-time contrasts in total).

To standardize interpretation across outcomes, where lower scores typically indicate improvement (eg, reduced mental distress), we set the aPASAT group as the reference. In this coding scheme, positive interaction effects indicate greater improvement in the aPASAT group relative to the SOR group. For outcomes where higher scores reflect improvement (eg, cognitive control and adaptive emotion regulation), the same reference coding applies, and negative interaction effects similarly indicate greater improvement in the aPASAT group. Two-tailed *P* values for fixed effects were calculated using the *lmerTest* package (version 3.1‐3) [88], applying restricted maximum likelihood estimation and Satterthwaite approximation for degrees of freedom [89]. Effect sizes for fixed effects were computed by dividing the beta coefficient by the SD of outcome scores post training in the SOR condition [90]. CIs (95%) were derived using nonparametric bootstrapping with 1000 resamples [91].

To evaluate the robustness of results against missing data, we used a pattern-mixture model approach [92]. To this end, we compared pattern-mixture models including missingness as an additional fixed effect to standard models via likelihood ratio tests.

To explore potential moderators, we tested 3-way interactions between each moderator, time of assessment, and condition. Moderators included training progress (measured as change in median ISI from first to last training session [41]), number of completed training sessions, and participant age (given known age-related decline in cognitive control; see [93]). All moderators were z-standardized.

Finally, we conducted a per-protocol analysis including only participants who completed at least 7 or more (ie, ≥70%) training sessions and computed proportions of clinically significant change following the criteria by Jacobson and Truax [94].

### Ethical Considerations

This monocenter randomized controlled superiority trial with 2 parallel arms was approved by the ethics committee of the Georg-Elias-Müller-Institute of Psychology, University of Göttingen, Germany (case number #205) and registered at the German Clinical Trials Register [95]. The study procedures adhered to the ethical standards of the institutional and national research committees and to the principles of the Declaration of Helsinki. All participants provided written informed consent and received financial compensation (€50 [approximately US $55]) for their participation. All data were deidentified prior to analysis to ensure participant privacy and confidentiality. Deidentified data, analysis code, and source code of the mobile trainings and assessments are available on the project’s Open Science Framework page [67].

## Results

### Participant Overview

Sample characteristics including the distribution of diagnoses across conditions and the total sample can be seen in Table 1. Training groups did not differ regarding any of these characteristics (all *P*>.24).

**Figure 1.**
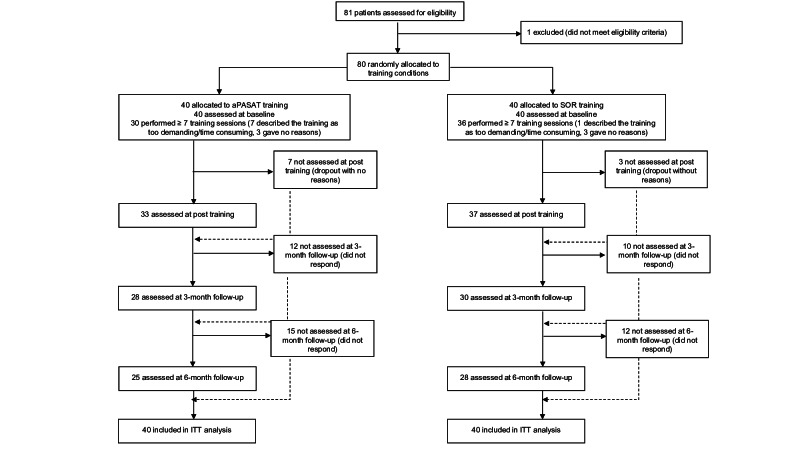
CONSORT (Consolidated Standards of Reporting Trials) flowchart*.* aPASAT: adaptive version of the Paced Auditory Serial Addition Test; ITT: intention-to-treat; SOR: Speed of Response Task.

Participant flow is displayed in Figure 1. Fifteen (38%) patients in the cognitive control training group did not complete the 6-month follow-up, and 12 (30%) in the speed of response training group. Rates of complete and incomplete assessments did not differ (*χ*^2^_1_=0.81; *P*=.37).

Participants in the cognitive control training group performed 8.1 training sessions on average (SD 2.9). Participants in the speed of response training condition performed 9.1 (SD 1.8) training sessions on average. Training dosage did not differ between groups (*t*_62.15_=1.73; *P*=.09). Thirty (75%) participants in the cognitive control group completed the training per protocol (ie, at least 7 training sessions), and 36 (90%) in the speed of response training group. Rates of training completion per protocol again did not differ (*χ*^2^_1_=2.16; *P*=.14).

On average, participants in the cognitive control training group spent 14.0 (SD 9.9) days completing the training sessions, while participants in the speed of response training group spent 15.5 (SD 7.7) days. Training groups did not differ in this regard (*t*_71.61_=0.76; *P*=.45).

At posttraining assessment, 8 patients in the aPASAT training condition had started outpatient therapy, while 5 did so in the SOR training group. Furthermore, patients in the aPASAT training group received, on average, 3.1 (SD 1.9) therapy sessions until posttraining assessment, and patients in the SOR group received 2.8 (SD 1.3) sessions. At the 3-month follow-up, 26 patients in the aPASAT and 35 in the SOR training condition commenced psychotherapy. Patients in the aPASAT group received, on average, 7.2 (SD 3.0) sessions until 3-month follow-up, while patients in the SOR group received 7.5 (SD 3.1) sessions. Finally, at the 6-month follow-up, nearly all participants have started the awaited outpatient psychotherapy, with 29 in the aPASAT and 36 in the SOR training group. Patients in the aPASAT group received 11.0 (SD 6.4) therapy sessions until 6-month follow-up, while patients in the SOR group received 11.5 (SD 4.5) sessions. Training groups did not differ in therapy commencement (all *P*>.46), nor in the number of therapy sessions at any assessment (all *P*>.72).

Means, SDs, and number of participants for primary, secondary, and disorder-specific outcomes of depression and (social) anxiety across all assessments can be found in Table 2. Due to the small number of patients meeting criteria for a diagnosis of posttraumatic stress disorder, somatic symptom disorder, borderline personality disorder, an eating disorder, obsessive-compulsive disorder, substance use disorder, or adjustment disorder (each <5, see Table 1), no statistical models on disorder-specific measures have been conducted for these subsamples. Still, means, SDs, and number of participants for these outcomes together with quality of life and work and social adjustment across all assessments are available in Table S4 in Multimedia Appendix 1.

**Table 2.** Means and SDs at baseline, post training, and follow-ups.

Outcome	Baseline		Post training		Three-month follow-up		Six-month follow-up	
	aPASAT^a^	SOR^b^	aPASAT	SOR	aPASAT	SOR	aPASAT	SOR
HSCL-11^c^								
n	40	40	33	37	28	30	25	28
Mean (SD)	2.07 (0.57)	2.00 (0.50)	2.03 (0.56)	1.87 (0.59)	1.98 (0.53)	1.79 (0.51)	1.92 (0.51)	1.86 (0.52)
RSQ-10D^d^								
n	40	40	32	37	28	30	25	28
Mean (SD)	23.27 (5.30)	23.30 (4.88)	23.50 (4.33)	21.95 (5.47)	23.36 (5.21)	20.77 (4.92)	22.48 (5.61)	21.11 (5.43)
PTQ^e^								
n	40	40	32	37	28	30	25	28
Mean (SD)	39.55 (9.75)	36.17 (10.05)	39.19 (7.91)	31.78 (11.84)	35.37 (8.83)	31.30 (11.03)	35.40 (10.92)	32.50 (12.85)
DERS-16^f^								
n	40	40	32	37	28	30	25	28
Mean (SD)	51.00 (13.50)	44.88 (11.61)	49.66 (11.24)	45.16 (14.00)	46.07 (11.13)	41.93 (12.54)	48.24 (12.29)	42.93 (14.90)
CERQ^g^ (adaptive)								
n	40	40	32	37	28	30	25	28
Mean (SD)	49.55 (9.07)	55.90 (11.95)	51.47 (9.39)	56.19 (12.94)	51.14 (9.10)	53.67 (12.33)	50.56 (8.05)	51.82 (12.33)
CERQ^h^ (maladaptive)								
n	40	40	32	37	28	30	25	28
Mean (SD)	39.64 (9.31)	38.80 (8.71)	40.44 (9.10)	37.78 (10.52)	39.11 (7.71)	36.67 (10.38)	39.60 (9.83)	36.75 (12.72)
PASAT^i^								
n	37	36	33	36	25	27	24	23
Mean (SD)	0.58 (0.18)	0.56 (0.16)	0.88 (0.12)	0.71 (0.15)	0.87 (0.09)	0.73 (0.14)	0.90 (0.06)	0.79 (0.13)
PHQ-9^j^								
n	31	34	25	31	22	26	18	24
Mean (SD)	12.94 (5.08)	11.24 (5.07)	11.08 (4.66)	9.50 (5.25)	11.32 (3.93)	8.92 (4.17)	10.78 (4.10)	9.50 (4.42)
GAD-7^k^								
n	15	16	12	15	10	12	10	12
Mean (SD)	10.07 (5.13)	8.62 (4.81)	9.92 (4.19)	7.93 (4.22)	6.10 (3.75)	6.83 (4.09)	8.50 (4.28)	8.75 (5.12)
mini-SPIN^l^								
n	10	11	7	11	9	8	8	6
Mean (SD)	9.80 (2.39)	7.09 (2.81)	10.29 (1.80)	6.18 (3.06)	8.56 (2.79)	7.25 (2.60)	9.00 (2.73)	7.83 (3.06)
RSQ-10D^m^ (brooding)								
n	40	40	32	37	28	30	25	28
Mean (SD)	11.15 (2.82)	11.07 (2.64)	11.16 (2.13)	10.62 (2.86)	11.04 (2.74)	10.10 (2.62)	10.84 (2.82)	10.04 (2.81)
RSQ-10D^n^ (reflection)								
n	40	40	32	37	28	30	25	28
Mean (SD)	12.12 (3.17)	12.22 (2.84)	12.34 (2.60)	11.32 (3.00)	12.32 (2.87)	10.67 (2.71)	11.64 (2.97)	11.07 (2.96)

a aPASAT: adaptive version of Paced Auditory Serial Addition Test.

b SOR: Speed of Response Task.

c HSCL-11: Hopkins Symptom Checklist-11.

d RSQ-10D: Response Style Questionnaire (10-item version).

e PTQ: Perseverative Thinking Questionnaire.

f DERS-16: Difficulties in Emotion Regulation Questionnaire (16-item version).

g CERQ (adaptive): Cognitive Emotion Regulation Questionnaire (adaptive subscale).

h CERQ (maladaptive): Cognitive Emotion Regulation Questionnaire (maladaptive subscale).

i PASAT: Paced Auditory Serial Addition Test.

j PHQ-9: Patient Health Questionnaire 9.

k GAD-7: Generalized Anxiety Disorder Scale 7.

l mini-SPIN: Mini-form of the Social Phobia Inventory.

m RSQ-10D (brooding): Response Style Questionnaire (brooding subfacet).

n RSQ-10D (reflection): Response Style Questionnaire (reflection subfacet).

### Intention-to-Treat Analysis

#### Global Mental Distress

**Figure 2.**
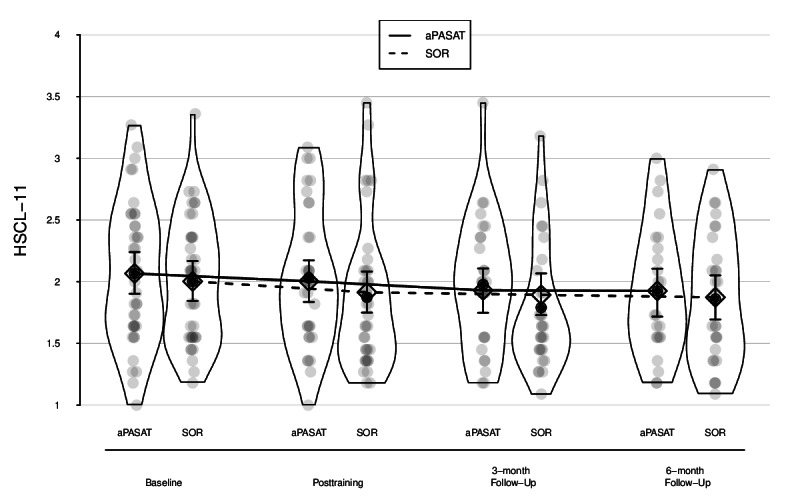
Model-based change trajectories in global mental distress. Gray dots = individual data points; black dots = empirical means; diamonds = model−based estimates (95% CIs). Bean width reflects distribution. aPASAT: adaptive Paced Auditory Serial Addition Test training; HSCL−11: Hopkins symptom checklist 11; SOR: Speed of response training.

The full-reduced model comparison for the primary outcome indicated that including training condition and its interaction with time of assessment did not significantly improve the model fit for global mental distress (*χ*^2^_4_=0.60; *P*=.96). Consistent with the full-reduced model comparison, the individual effect for the interaction between training condition and time until postassessment (primary end point) was also not significant (B=−0.03, 95% CI −0.21, 0.16; *t*_179.60_=−0.26; *P*=.80; *d*=−0.04, 95% CI−0.35, 0.28). The same applied to the interaction effect of training condition and time between baseline and 3-month as well as 6-month follow-up assessment (secondary end point; B=0.03, 95% CI −0.17, 0.23; *t*_181.34_=0.25; *P*=.80; *d*=0.05, 95% CI −0.28, 0.39; and B=0.01, 95% CI −0.21, 0.22; *t*_181.69_=0.11; *P*=.91; *d*=0.02, 95% CI −0.35, 0.37, respectively). Taken together, this indicates that the change in global mental distress over time was not different between training groups, neither in the short term nor in the long term. Figure 2 displays the change trajectories for HSCL-11 scores by group, including both raw data and model-based estimates. In addition, Figure S3 in the Multimedia Appendix 1 depicts individual patient-level changes in global mental distress as measured with the HSCL-11 from baseline to 6-month follow-up.

#### Cognitive Control

The full-reduced model comparison indicated that including training condition and its interaction with time of assessment significantly improved model fit for the PASAT assessment outcome (*χ*^2^_4_=389.04; *P*<.001). Consistent with this, the individual effect for the interaction between training condition and time until posttraining assessment was significant for PASAT performance (B=−0.56, 95% CI −0.59,‐0.54; *z*=–18.02; *P*<.001; *d*=−1.23, 95% CI −1.30,−1.20). The same was true for the interaction between training condition and time until 3- and 6-month follow-up (B=−0.42, 95% CI −0.48,−0.40; *z*=−11.62; *P*<.001; *d*=−0.92, 95% CI −1.06,−0.88; and B=−0.44, 95% CI −0.50,−0.42; *z*=−11.22; *P*<.001; *d*=−0.97, 95% CI −1.10,−0.92, respectively). In summary, the improvement in cognitive controls as measured by the assessment version of the PASAT was more pronounced in the cognitive control training group across all points in time.

#### Emotion Regulation

None of the full-reduced model comparisons for the outcomes measuring rumination, repetitive negative thinking, difficulties in emotion regulation, or cognitive emotion regulation were significant (all *P* >.06). This indicates that overall changes over time were not different between training conditions for these measures. Consequently, significant effects at the level of individual interactions (eg, the interaction between training condition and time from baseline to posttraining assessment) should be interpreted with caution, as we did not specify hypotheses for every outcome at each assessment, and the effects were not strong enough to substantially improve model fit beyond what was explained by time alone considering all assessments.

However, the analysis of time point–specific interaction effects indicated that cognitive control training was superior to speed of response training in improving difficulties in emotion regulation (B=4.73, 95% CI 0.52, 9.12; *t*_177.99_=2.09; *P*=.04; *d*=0.34, 95% CI 0.04, 0.65) and adaptive cognitive emotion regulation (B=−3.81, 95% CI −8.55, 0.67; *t*_180.51_=–1.64; *P*=.10; *d*=−0.29, 95% CI −0.66, 0.05) from baseline to 6-month follow-up. In contrast, cognitive control training appeared less efficacious than the speed of response training in reducing rumination over the same period (B=−2.35, 95% CI −4.49,–0.19; *t*_182.49_=−2.16; *P*=.03; *d*=−0.43, 95% CI −0.82,−0.03). Details on estimates and effect sizes for other nonsignificant comparisons as well as conditional main effects of time (effects of time in the cognitive control training group) can be found in Table S3 in Multimedia Appendix 1.

#### Disorder-Specific Outcomes

##### Depression

Most patients (81%) met criteria for a diagnosis of major depressive disorder (see Table 1). As indicated by the nonsignificant full-reduced model comparison, training groups did not differ regarding changes in symptoms of depression as measured with the PHQ-9 (*χ*^2^_4_=3.08; *P*=.54). Consistent with that, symptoms of depression did not change differently between training groups from baseline to posttraining assessment (B=0.85, 95% CI −0.92, 2.74; *t*_142.00_=0.87; *P*=.39; *d*=0.16, 95% CI −0.18, 0.52), 3-month follow-up (B=1.26, 95% CI −0.53, 3.14; *t*_143.30_=1.21; *P*=.23; *d*=0.24, 95% CI −0.10, 0.60), or 6-month follow-up (B=1.39, 95% CI −0.65, 3.57; *t*_143.72_=1.27; *P*=.21; *d*=0.26, 95% CI −0.12, 0.68).

##### Social Anxiety

The full-reduced model comparisons revealed a significant interaction of time and training condition for symptoms of social anxiety as measured by the mini-SPIN (*χ*^2^_4_=13.32; *P*=.01). While symptoms of social anxiety did not change differently between training groups from baseline to posttraining assessment (B=−0.32, 95% CI −1.93, 1.24; *t*_43.04_=−0.37; *P*=.72; *d*=−0.10, 95% CI −0.63, 0.41), the interaction was significant from baseline to 3-month follow-up (B=1.93, 95% CI 0.28, 3.72; *t*_43.26_=2.16; *P*=.04; *d*=0.63, 95% CI 0.09, 1.21), and from baseline to 6-month follow-up (B=2.00, 95% CI 0.14, 3.81; *t*_43.43_=2.08; *P*=.04; *d*=0.66, 95% CI 0.05, 1.24). However, these findings are based on the small number of patients who met the diagnostic criteria for SAD and who had also completed the 3-month follow-up (n=17) or 6-month follow-up (n=14), respectively (see Table S6 in Multimedia Appendix 1 for the number of measurements per outcome and assessment). As such, they should be interpreted with caution.

##### Anxiety

The full-reduced model comparison showed that the interaction between training condition and time did not improve the model fit for symptoms of anxiety other than social phobia beyond effects of time (*χ*^2^_4_=5.43; *P*=.25). In line with the full-reduced model comparison, symptoms of anxiety did not change differently between groups from baseline to posttraining assessment (B=0.45, 95% CI −1.90, 2.99; *t*_66.00_=0.34; *P*=.73; *d*=0.11, 95% CI −0.45, 0.71). However, the interaction of training condition and time was significant between baseline and 3-month follow-up (B=2.94, 95% CI 0.38, 5.82; *t*_66.51_=2.09; *P*=.04; *d*=0.70, 95% CI 0.09, 1.38), but this effect was not maintained to the 6-month follow-up (B=1.65, 95% CI −0.91, 4.32; *t*_66.46_=1.17; *P*=.25; *d*=0.39, 95% CI −0.22, 1.03). Like the analysis for symptoms of social anxiety, it was only a small number of patients (n=22) who met the diagnostic criteria for an anxiety disorder other than SAD and who also completed the 3-month follow-up (see Table S6 in Multimedia Appendix 1). As with the analysis of emotion regulation, the nonsignificant full-reduced model comparison advises caution in interpreting single interaction effects.

All other disorder-specific outcomes were not analyzed in detail due to the small number of patients (each n<5) meeting diagnostic criteria of the respective disorder. However, means and SDs for these outcomes are provided in Table S4 in Multimedia Appendix 1.

##### Quality of Life and Work and Social Adjustment

All full-reduced model comparisons showed that adding training condition and its interaction with time did not change the model fit for any quality of life and work and social adjustment outcome, including all WHOQOL-BREF subscales and the WSAS, all *P*>.22. Estimates and effect sizes can be found in Table S3 in Multimedia Appendix 1.

##### Additional Tables and Figures

The number of measurements and participants in the linear mixed-effect models can be found in Table S5 in Multimedia Appendix 1. Change trajectories for other outcomes, including disorder-specific outcomes and quality of life and work and social adjustment, are illustrated in Figures S4-S17 in Multimedia Appendix 1.

### Credibility and Expectancy

Credibility and expectancy for the training’s efficacy both decreased between baseline and posttraining assessment (B=−0.82, *t*_77.75_=–2.79, *P*=.007; and B=–0.63, *t*_74.68_=–2.34, *P*=.02, respectively). Groups did not differ in this regard (all *P*>.34).

### Clinical Significance

Only 1 patient experienced a clinically significant change in the primary outcome (decrease in HSCL-11 total score of at least 0.62 and final HSCL-11 score lower than 1.91) [68] from baseline to 6-month follow-up. No clinically significant changes occurred from baseline to the posttraining or 3-month follow-up assessment. Additionally, there were no patients who experienced a clinically significant change in symptoms of depression (decrease in PHQ-9 of at least 6.76 and final PHQ-9 score below 8.60) [80] from baseline to post training, 3-month, or 6-month follow-up assessment.

### Missing Data Analysis

For all measures including global mental distress, emotion regulation, and cognitive control, likelihood ratio tests of the pattern mixture models indicated that the results did not change when including missingness into the models (all *P*>.28, see Table S6 in Multimedia Appendix 1). This indicates that missing values had no influence on the above-reported results.

### Per-Protocol Analysis

The analysis of the data of participants that finished at least two-thirds of the training sessions revealed the same pattern of results with one exception: for repetitive negative thinking, the full-reduced model comparison was significant, *P*=.05. However, none of the individual interaction effects between training condition and time of assessment between baseline, post training, 3-, or 6-month follow-up were significant (all *P*>.19). Therefore, the significant full-reduced model comparison likely reveals a general difference between training conditions in repetitive negative thinking, which did not change across time of assessments.

For detailed estimates and effect sizes for direct comparisons between training groups at all assessments in the per-protocol analysis, see Table S7 and Table S8 for the number of measurements and participants in Multimedia Appendix 1. Figures S18-S24 in Multimedia Appendix 1 depict change trajectories for global mental distress, emotion regulation, and cognitive control across conditions in the per-protocol analysis.

### Moderation Analysis

#### Training Progress

Training performance as measured by the median ISI in a training session improved in the cognitive control training condition (B=–64.70; *t*_69.28_=–7.60; *P*<.001). A significant interaction effect showed that the training progress was stronger in the cognitive control compared to the speed of response training group (B=57.31; *t*_68.56_=4.94; *P*<.001).

Training progress as measured by change in median ISI significantly moderated the interaction effect between training condition and time for the performance in the assessment version of the PASAT (B=–0.08; *z*=–5.65; *P*<.001). This simply indicates that cognitive control training was more efficacious in improving near-transfer cognitive control for those patients with more training progress. All other moderation effects by training progress were not significant (all *P*>.12).

#### Age

The 3-way interaction between age, training condition, and assessment was not significant for any primary or secondary outcome (all *P*>.09), indicating that differing changes in outcomes between training groups over time were not moderated by the age of participants.

#### Number of Training Sessions

Although the number of completed training sessions was not different between training conditions (see above), the number of completed training sessions significantly moderated the interaction effect of time and training condition. Specifically, completing more training sessions led to greater reductions in global mental distress in the speed of response compared to the cognitive control training (B=–0.16; *t*_204.86_=–3.01; *P*=.003). The same was true for improvements in rumination (B=–1.64; *t*_208.79_=–3.02; *P*=.003) and cognitive control (B=–0.18; *z*=–7.68; *P*<.001). In other words, participants who received the speed of response training showed greater improvements in these outcomes than those in the cognitive control group when they completed more training sessions.

## Discussion

### Principal Results

To our knowledge, this study is the first to examine the efficacy of mobile cognitive control training across a broad spectrum of mental disorders in individuals waiting for outpatient psychotherapy. Contrary to our primary hypothesis, cognitive control training, using the adaptive PASAT, was not superior to speed of response training in reducing global mental distress, neither in the short nor in the long term.

### Comparison With Prior Work

These findings diverge from earlier research on adaptive PASAT training in individuals with major depression [39-43]. Four of these 5 studies focusing on patients with current major depression combined cognitive control training with transcranial direct current stimulation [39,40,42,43]. Notably, control groups in these studies receiving cognitive control training paired with sham transcranial direct current stimulation improved as well, suggesting that cognitive control training might be similarly effective without any parallel brain stimulation. However, these studies were characterized by relatively small sample sizes, with the largest n=43 [41]. This limitation is notable, as only large effects could be detected in such small samples, unlike the smaller overall effect (Hedges *g*=0.29) reported for PASAT-based cognitive control training in a meta-analysis [48]. Consequently, studies [39] and [42], which reported large postassessment training effects greater than 1.00, may be considered outliers given their small sample sizes. When planning our study, we based our sample size on the most comparable study using the same intervention protocol, which reported a medium-sized effect on the primary outcome [45]. However, considering the meta-analysis published after our data collection was completed, future studies should aim for larger sample sizes. Our results are consistent with studies that examined cognitive control training as an adjunct to inpatient treatment [55] or in combination with behavioral activation for outpatients [54], which found no added benefit from the training. Given the efficacy of cognitive control training in studies with larger samples of individuals with vulnerability to or in remission from major depression [45,96], it can be tentatively concluded that the intervention may be more suitable as a (relapse) prevention for depression and less for people with an acute full-syndrome mental disorder.

### Implications

The cognitive control training led to significant improvements in the nonadaptive assessment version of the PASAT, showing a near-transfer effect. This outcome is expected given that the training and the assessment version of the task are almost identical. Cognitive control training often involves assessment tasks closely related to those used in the intervention, and studies typically report limited far-transfer effects to other cognitive tasks [53,55,97]. Conversely, one study found that adaptive PASAT training enhanced performance in an n-back task, which targets similar cognitive functions, highlighting some degree of transfer [49]. Given the already challenging nature of the PASAT [98,99] and the number of outcome measures used to cover a broad spectrum of clinical parameters and emotion regulation, we did not assess transfer effects to other cognitive control tasks. Despite several months without further training, the cognitive control training group maintained a higher performance on the assessment version of the PASAT compared to the speed of response group at follow-ups, indicating the potential for a lasting impact of the training. Future research should explore far-transfer effects using various cognitive measures, ideally with minimal overlap in processes not related to cognitive control, such as auditory or spatial processing.

The analysis of individual effects indicated superiority of the cognitive control training in improving difficulties in emotion regulation from baseline to 3-month follow-up and in cognitive emotion regulation from baseline to 6-month follow-up, respectively. These results align with prior research on patients remitted from depression [45]. However, contrary to the findings in the previous study that also used the CERQ, our results showed an interaction effect in the compound score for adaptive cognitive emotion regulation. We did not find any differences for the compound score of maladaptive cognition and emotion regulation. Additionally, a significant interaction effect for rumination from baseline to 3-month follow-up showed that the improvement was stronger for the speed of response training group. This result, which indicates the inferiority of the PASAT training condition regarding rumination, is in stark contrast to the meta-analytic findings in clinically depressed samples [48]. However, for all outcomes related to emotion regulation, the full-reduced model comparison was not significant. Therefore, these single effects were not substantial enough considering all assessments. As we did not formulate hypotheses for each of these secondary outcomes for each given time interval, there is a risk for these individual effects to be statistical artifacts, and they should be interpreted with caution.

The findings on disorder-specific symptoms tentatively suggest that the training may be able to reduce anxiety symptoms, especially social anxiety, in the long term. However, it is important to consider the limited number of patients with the respective diagnosis who provided data at follow-up assessments (see Table S5 in Multimedia Appendix 1). Thus, these findings should be treated with caution until replication in larger samples.

Any improvements in outcomes that were not significantly different between training conditions may be attributed to factors that applied to both groups equally, such as the commencement of or number of psychotherapy sessions during follow-up periods (which were not different between training groups), spontaneous symptom improvements, regression to the mean, demand effects, or a combination of those. It also cannot be ruled out that shared but unknown mechanisms of both cognitive control and speed of response training contributed to these improvements. Although the speed of response task may not train the updating of working memory content, it does necessitate sustained attention to effectively perform the task, thus making it an active control condition [45].

Finally, the optimal dosage and frequency of cognitive control training need to be established. A recent study suggests that at least 10 training sessions are necessary to achieve a medium-sized effect on symptoms of depression [100]. Consistent with our approach, most studies using the PASAT have implemented approximately 10 training sessions [48]. Contrary to our expectations, the moderation analysis suggests that cognitive control training was relatively less efficacious than speed of response training in alleviating global mental distress, reducing rumination, and improving cognitive control among participants who completed a high number of training sessions. However, this was an exploratory analysis, and identifying moderators in 3-way interactions typically requires sample sizes in the several hundred [101], far exceeding that of this study. Moreover, the number of training sessions was limited, as participants could complete 10 training sessions at most. Additionally, most participants completed 7 or more training sessions (see “Participant Overview”). This limits the variability needed to meaningfully assess the moderating role of differing numbers of training sessions.

Beyond determining the optimal dosage for posttraining effects, future research should address whether booster sessions during follow-up periods could maintain or enhance long-term outcomes. Furthermore, it would be similarly important to investigate the ideal timing of training sessions, for instance, in individuals at risk for depression, training during periods of increased rumination may amplify its efficacy [102]. In this regard, mobile health interventions, such as the mobile cognitive control training studied here, might serve as suitable just-in-time adaptive interventions [103].

### Limitations

The findings of this study should be interpreted considering its methodological strengths and limitations. Despite our efforts to reach out to patients to encourage the completion of assessments, nearly half of the participants had missing data at one of the 4 assessments, primarily the 3-month and 6-month follow-up (see Figure 1). Notably, however, attrition rates did not differ between groups, and the vast number of participants finished the training per protocol, that is, they completed at least 7 of the 10 scheduled training sessions. This indicates that factors related to attrition at follow-up are likely unrelated to the specifics of the training protocol. Moreover, our follow-up sample sizes are comparable with those in other cognitive control training studies [48].

While a meta-analysis suggests that PASAT-based cognitive control training is more effective in reducing depressive symptoms immediately after the training than at follow-up [48], the lack of follow-up assessments in most studies limits conclusions about long-term effects. Small samples and highly variable follow-up intervals (ranging from weeks to a year) further reduce power and comparability. Some evidence points to longer-term benefits for outcomes such as rumination and emotion regulation [45]. These effects may unfold gradually, as participants need time to apply trained strategies such as filtering irrelevant (negative) information from working memory in daily life. Future studies should extend follow-up periods up to 12 months, as done in cognitive bias modification research [104], and explore whether training-induced gains in cognitive control enhance responsiveness to subsequent psychotherapy [105].

Our sample, with a mean age of 25.8 (SD 5.2) years, was relatively young compared to other cognitive control training studies in depression [48]. This might be since the treatment facility is situated on a university campus, and therefore often frequented by students with mental health problems. Cognitive control tends to decline with age [93], but we found no evidence that age moderated training effects in our relatively young sample. Future studies should include more diverse community samples in terms of age and education. Since mental disorders frequently co-occur, some participants met criteria for more than one disorder (see Table 1). This raises the possibility of overlap in disorder-specific analyses, as individuals may influence multiple outcomes. However, our primary outcome was global mental distress, not tied to any single diagnosis. Given the high prevalence of comorbidity in clinical populations [106], we chose to include patients with multiple diagnoses to enhance ecological validity. Finally, due to the monocentric design and recruitment from a Western European outpatient setting, the generalizability of our findings to other clinical or cultural contexts may be limited.

Notably, almost no patients showed clinically significant improvements in global mental distress or depressive symptoms at the 6-month follow-up, which is unexpected given that most began psychotherapy during this period. However, our sample reported relatively low baseline distress, leaving limited room for substantial improvement. For example, patients with major depression had an average baseline PHQ-9 of 12.0 (see Table 2). Given the PHQ-9’s reliable change index of 6.8 points [80], a clinically significant improvement would require a follow-up score below 6, which is typical of healthy individuals [107]. Moreover, such changes generally do not occur within the first few sessions of outpatient therapy [108]. That most participants began psychotherapy during the follow-up is unsurprising given the recruitment from a waiting list. While this could confound observed improvements, clinically significant changes were rare overall, and psychotherapy uptake did not differ between training groups, nor did the number of therapy sessions between groups. Thus, any confounding effect by psychotherapy is either likely minimal or evenly distributed between groups.

### Conclusions

This study examined the efficacy of mobile cognitive control training as a transdiagnostic intervention for individuals awaiting outpatient psychotherapy. Despite its limitations, the study’s design allowed for an ecologically valid training context. In summary, we did not find evidence for the superiority of the cognitive control training over an active control in reducing symptoms of mental distress. Based on our results, we cannot generally suggest cognitive control training in its current form as an intervention for patients with an acute, full-blown mental disorder. Furthermore, examining the phase of illness, potential working mechanisms, and far-transfer effects in large samples is necessary to further assess the potential of this intervention.

## Supplementary material

10.2196/65867 Multimedia Appendix 1 Additional material including tables, figures, and details for disorder-specific outcomes.

10.2196/65867 Checklist 1 CONSORT checklist.

## Abbreviations

aPASAT: adaptive version of the PASA
CEQ: Credibility Expectancy Questionnaire
CERQ: Cognitive Emotion Regulation Questionnaire
CONSORT: Consolidated Standards of Reporting Trials
DERS-16: Difficulties in Emotion Regulation Scale 16
*DSM-5*: *Diagnostic and Statistical Manual of Mental Disorders* (Fifth Edition)
GAD-7: Generalized Anxiety Disorder Questionnaire
HSCL-11: Hopkins Symptom Checklist-11
ISI: interstimulus interval
mini-SPIN: Mini-Social Phobia Inventory
PASAT: Paced Auditory Serial Addition Test
PHQ-9: Patient Health Questionnaire 9
PTQ: Perseverative Thinking Questionnaire
RDoC: Research Domain Criteria
RSQ: Response Style Questionnaire
SAD: Social anxiety disorder
SOR: Speed of Response Task
WHOQOL-BREF: World Health Organization Quality of Life Brief Form
WSAS: Work and Social Adjustment Scale
