# Supplementary material for: Transdiagnostic Cognitive Control Training for Patients Waiting for Outpatient Psychotherapy: Randomized Clinical Trial
Source: JMIR Mhealth Uhealth. 2025 Nov 26;13:e65867. doi: 10.2196/65867 (PMC12655892; doi:10.2196/65867)
Supplement: Multimedia Appendix 1 [file mhealth-v13-e65867-s001.pdf]

Supplement to the Study “Transdiagnostic Cognitive Control Training for Patients waiting for Outpatient Psychotherapy: A Randomized Clinical Trial”

Disorder-specific questionnaires

Symptoms of Depression. For those patients that met DSM-5 criteria for a diagnosis of major depressive disorder, symptoms of depression were measured by the *Patient Health Questionnaire 9* (PHQ-9; [1]). The PHQ-9 is a commonly used self-report questionnaire with good psychometric properties [1] and sensitivity to change [2]. It comprises nine items which assess depressive symptoms on a 4-point Likert scale (total scores range between 0 and 27). At baseline, Cronbachs’s alpha was 0.80.

Symptoms of Anxiety. Symptoms of anxiety in patients with an anxiety disorder (excluding SAD) were assessed using the *Generalized Anxiety Disorder Questionnaire* (GAD-7; [3]). The GAD-7 measures symptoms of anxiety on a 4-point Likert scale. Total scores range between 0 and 21. The GAD-7 has shown to be a psychometrically sound measure [3] that can reliably detect symptoms of various anxiety disorders [4,5]. At baseline, Cronbachs’s alpha was 0.86.

Symptoms of Social Anxiety. Symptoms of social anxiety were assessed using the *Mini-Social Phobia Inventory* (mini-SPIN; [6]). The mini-SPIN is a 3-item condensed version of the original *Social Phobia Inventory* [7] to target core aspects of social anxiety, including fear of embarrassment, avoidance of social situations, and distress in social situations. Items are scored on a Likert scale from 0 to 4. Higher scores indicate stronger symptoms of social phobia. Psychometric reliability and validity for the mini-SPIN have been reported [6]. At baseline, Cronbachs’s alpha was 0.72.

Other disorder-specific measures. Disorder-specific outcomes for less common diagnoses were assessed with self-report questionnaires. Due to the small number of affected participants (see Table 1), internal consistency was not calculated.

Symptoms of somatic symptom disorder were assessed using the *Patient Health Questionnaire-15* (PHQ-15; [8]), a self-report questionnaire that maps the severity and psychological distress due to somatic symptoms. Total scores range between 0 and 30, with higher scores indicative of more distress.

Patients with an eating disorder filled in the *Eating Disorder Examination Questionnaire 8* (EDE-Q8; [9]), a brief self-report measure to assess the range and severity of eating disorder symptoms, such as restraint, eating concern, shape concern, and weight concern. Total mean scores range between 0 and 6, with higher scores indicative of more severe symptoms.

Symptoms associated with Borderline Personality Disorder (BPD) were assessed with the *Borderline Symptom List 23* (BSL-23; [10]), a concise self-report questionnaire intended to provide a quick and efficient means of evaluating core BPD symptoms such as affective instability, identity disturbance, negative relationships, and self-harm behaviors. Total sum scores range between 0 and 92, with higher scores indicative of more severe symptoms.

Patients with a substance use disorder, filled in the *Mannheimer Craving Scale* (MaCS; [11]), a measure of craving intensity, capturing various dimensions, including its strength, frequency, and duration, as well as the emotional and physical sensations associated with craving episodes. Total scores range between 0 and 48, with higher scores indicative of more craving.

Symptoms of adjustment disorder were assessed using the *Adjustment Disorder – New Module* (ADNM-20; [12]), a questionnaire developed to identify and quantify the severity of symptoms in individuals experiencing significant emotional or behavioral symptoms in response to identifiable stressors, within three months of the stressor occurring. Total scores range between 20 and 80, with higher scores indicative of more distress.

Symptoms of PTSD were assessed in individuals with the respective diagnoses using the *Short Screening Scale for PTSD* (PTSD-7; [13]). It consists of 7 items that assess key symptoms of PTSD, including re-experiencing the traumatic event, avoidance of trauma-related stimuli, negative alterations in cognition and

mood, and alterations in arousal and reactivity. Total sum scores range between 0 and 27, with higher scores indicative of more severe symptoms.

Symptoms associated with OCD were measured with the *Obsessive-Compulsive Inventory-Revised* (OCI-R; [14]). The OCI-R comprises 18 items evaluating various dimensions of OCD, including washing, checking, ordering, obsessing, hoarding, and mental neutralizing. Total sum scores range from 0 to 7, with higher scores indicative of more severe symptoms.

Supplement References

1. Kroenke K, Spitzer RL, Williams JBW. The PHQ-9. Journal of General Internal Medicine. 2001 Sep;16(9):606–13.

2. Löwe B, Kroenke K, Herzog W, Gräfe K. Measuring depression outcome with a brief self-report instrument: Sensitivity to change of the Patient Health Questionnaire (PHQ-9). Journal of Affective Disorders. 2004 Jul;81(1):61–6.

3. Spitzer RL, Kroenke K, Williams JBW, Löwe B. A Brief Measure for Assessing Generalized Anxiety Disorder: The GAD-7. Archives of Internal Medicine. 2006 May;166(10):1092.

4. Kroenke K, Spitzer R, Williams J, Monahan P, Löwe B. Anxiety Disorders in Primary Care: Prevalence, Impairment, Comorbidity, and Detection. Annals of internal medicine. 2007 Apr;146:317–25.

5. Williams N. The GAD-7 questionnaire. Occupational Medicine. 2014 Apr;64(3):224–4.

6. Wiltink J, Kliem S, Michal M, Subic-Wrana C, Reiner I, Beutel ME, et al. Mini - social phobia inventory (mini-SPIN): Psychometric properties and population based norms of the German version. BMC Psychiatry. 2017 Nov;17(1):377.

7. Connor KM, Davidson JRT, Churchill LE, Sherwood A, Weisler RH, Foa E. Psychometric properties of the Social Phobia Inventory (SPIN): New self-rating scale. British Journal of Psychiatry. 2000 Apr;176(4):379–86.

8. Kroenke K, Spitzer RL, Williams JBW. The PHQ-15: Validity of a New Measure for Evaluating the Severity of Somatic Symptoms. Psychosomatic Medicine. 2002 Mar;64(2):258.

9. Kliem S, Mößle T, Zenger M, Strauß B, Brähler E, Hilbert A. The eating disorder examination-questionnaire 8: A brief measure of eating disorder psychopathology (EDE-Q8). International Journal of Eating Disorders. 2016;49(6):613–6.

10. Bohus M, Kleindienst N, Limberger MF, Stieglitz RD, Domsalla M, Chapman AL, et al. The Short Version of the Borderline Symptom List (BSL-23): Development and Initial Data on Psychometric Properties. Psychopathology. 2009;42(1):32–9.

11. Nakovics H, Diehl A, Geiselhart H, Mann K. Entwicklung und Validierung eines Instrumentes zur substanzunabhängigen Erfassung von Craving: Die Mannheimer Craving Scale (MaCS). Psychiatrische Praxis. 2009 Mar;36(2):72–8.

12. Lorenz L, Bachem R, Maercker A. The Adjustment Disorder–New Module 20 as a Screening Instrument: Cluster Analysis and Cut-off Values. The International Journal of Occupational and Environmental Medicine. 2016 Sep;7(4):215–20.

13. Siegrist P, Maercker A. Deutsche Fassung der Short Screening Scale for DSM-IV Posttraumatic Stress Disorder. Trauma & Gewalt. 2010;4(3):208–13.

14. Gönner S, Leonhart R, Ecker W. Das Zwangsinventar OCI-R - die deutsche Version des Obsessive-Compulsive Inventory-Revised. PPmP - Psychotherapie · Psychosomatik · Medizinische Psychologie. 2007 Jan;57(09/10):395–404.

Table S3

Estimates and Effect Sizes for Direct Comparisons Between Speed of Response and Adaptive Paced Serial Addition Test Training with 95% Confidence Interval

Outcome	Time (end-of-training)		Training x Time (end-of-training)		Time (3-month follow-up)		Training x Time (3-month follow-up)		Time (6-month follow-up)		Training x Time (6-month follow-up)	
	B	*d*	B	*d*	B	*d*	B	*d*	B	*d*	B	*d*
HSCL-11	-0.14<br>[-0.29, 0.00]	-0.14<br>[-0.29, 0.01]	-0.23<br>[-0.49, 0.01]	-0.24<br>[-0.50, 0.02]	-0.06<br>[-0.29, 0.18]	-0.03<br>[-0.21, 0.16]	-0.11<br>[-0.49, 0.31]	-0.04<br>[-0.35, 0.28]	0.03<br>[-0.17, 0.23]	0.01<br>[-0.21, 0.22]	0.05<br>[-0.28, 0.39]	0.02<br>[-0.35, 0.37]
RSQ-10D	-0.23<br>[-1.71, 1.24]	-1.12<br>[-2.65, 0.47]	-0.04<br>[-0.31, 0.23]	-0.20<br>[-0.49, 0.09]	0.03<br>[-2.22, 2.18]	-1.08<br>[-3.19, 0.86]	0.00<br>[-0.41, 0.40]	-0.20<br>[-0.58, 0.16]	-2.35<br>[-4.49, -0.19]	-1.37<br>[-3.58, 0.67]	-0.43<br>[-0.82, -0.03]	-0.25<br>[-0.66, 0.12]
PTQ	-4.60<br>[-7.63, -1.62]	-5.08<br>[-8.16, -2.00]	-0.39<br>[-0.64, -0.14]	-0.43<br>[-0.69, -0.17]	-3.37<br>[-7.57, 1.18]	-2.32<br>[-6.56, 1.72]	-0.29<br>[-0.64, 0.10]	-0.20<br>[-0.55, 0.15]	0.07<br>[-4.20, 4.28]	0.87<br>[-3.68, 5.27]	0.01<br>[-0.35, 0.36]	0.07<br>[-0.31, 0.45]
DERS-16	-6.53<br>[-9.81, -3.46]	-4.03<br>[-7.04, -0.57]	-0.47<br>[-0.70, -0.25]	-0.29<br>[-0.50, -0.04]	-6.13<br>[-11.47, -0.55]	3.51<br>[-0.66, 7.48]	-0.44<br>[-0.82, -0.04]	0.25<br>[-0.05, 0.53]	4.73<br>[0.52, 9.12]	1.55<br>[-3.07, 5.88]	0.34<br>[0.04, 0.65]	0.11<br>[-0.22, 0.42]
CERQ (adaptive)	2.13<br>[-1.14, 5.15]	2.53<br>[-0.79, 5.98]	0.16<br>[-0.09, 0.40]	0.20<br>[-0.06, 0.46]	6.35<br>[1.73, 10.90]	-1.83<br>[-6.06, 2.11]	0.49<br>[0.13, 0.84]	-0.14<br>[-0.47, 0.16]	-3.81<br>[-8.55, 0.67]	-5.18<br>[-9.74, -0.41]	-0.29<br>[-0.66, 0.05]	-0.40<br>[-0.75, -0.03]
CERQ (maladaptive)	-1.58<br>[-4.19, 0.95]	-1.63<br>[-4.26, 0.98]	-0.15<br>[-0.40, 0.09]	-0.15<br>[-0.40, 0.09]	-0.84<br>[-4.68, 3.13]	-0.87<br>[-4.38, 2.43]	-0.08<br>[-0.45, 0.30]	-0.08<br>[-0.42, 0.23]	-1.43<br>[-4.89, 2.05]	-1.21<br>[-5.21, 2.53]	-0.14<br>[-0.46, 0.19]	-0.11<br>[-0.50, 0.24]
PASAT	0.68<br>[0.69, 0.70]	0.76<br>[0.76, 0.77]	1.68<br>[1.70, 1.72]	1.85<br>[1.87, 1.89]	-0.03<br>[-0.17, -0.05]	-0.56<br>[-0.59, -0.54]	-0.08<br>[-0.41, -0.13]	-1.37<br>[-1.44, -1.33]	-0.42<br>[-0.48, -0.40]	-0.44<br>[-0.50, -0.42]	-1.02<br>[-1.18, -0.97]	-1.07<br>[-1.22, -1.02]
PHQ-9	-2.41<br>[-3.77, -0.96]	-2.66<br>[-4.23, -1.06]	-0.46<br>[-0.72, -0.18]	-0.51<br>[-0.81, -0.20]	-1.70<br>[-3.93, 0.49]	0.85<br>[-0.92, 2.74]	-0.32<br>[-0.75, 0.09]	0.16<br>[-0.18, 0.52]	1.26<br>[-0.53, 3.14]	1.39<br>[-0.65, 3.57]	0.24<br>[-0.10, 0.60]	0.26<br>[-0.12, 0.68]
GAD-7	-4.22<br>[-6.12, -2.16]	-1.82<br>[-3.73, 0.13]	-1.00<br>[-1.45, -0.51]	-0.43<br>[-0.89, 0.03]	-1.44<br>[-4.59, 1.51]	0.45<br>[-1.90, 2.99]	-0.34<br>[-1.09, 0.36]	0.11<br>[-0.45, 0.71]	2.94<br>[0.38, 5.82]	1.65<br>[-0.91, 4.32]	0.70<br>[0.09, 1.38]	0.39<br>[-0.22, 1.03]
mini-SPIN	-1.66<br>[-2.80, -0.52]	-0.97<br>[-2.15, 0.37]	-0.54<br>[-0.91, -0.17]	-0.32<br>[-0.70, 0.12]	-2.71<br>[-5.08, -0.53]	-0.32<br>[-1.93, 1.24]	-0.89<br>[-1.66, -0.17]	-0.10<br>[-0.63, 0.41]	1.93<br>[0.28, 3.72]	2.00<br>[0.14, 3.81]	0.63<br>[0.09, 1.21]	0.66<br>[0.05, 1.24]
WHOQOL-BREF (physical)	8.76<br>[4.08, 13.41]	8.71<br>[3.88, 13.38]	0.61<br>[0.28, 0.93]	0.60<br>[0.27, 0.93]	7.14<br>[0.22, 14.02]		0.49<br>[0.02, 0.97]		-3.45<br>[-10.90, 3.17]	-4.37<br>[-11.88, 1.89]	-0.24<br>[-0.75, 0.22]	-0.30<br>[-0.82, 0.13]
WHOQOL-BREF (psychological)	5.97<br>[1.77, 9.87]	3.04<br>[-1.53, 7.41]	0.35<br>[0.10, 0.57]	0.18<br>[-0.09, 0.43]	3.44<br>[-4.94, 10.76]		0.20<br>[-0.29, 0.62]		-4.41<br>[-10.30, 1.27]	1.98<br>[-4.23, 7.62]	-0.25<br>[-0.60, 0.07]	0.11<br>[-0.24, 0.44]
WHOQOL-BREF (social)	-1.75<br>[-8.21, 5.20]	-9.12<br>[-15.99, -1.98]	-0.09<br>[-0.40, 0.26]	-0.45<br>[-0.79, -0.10]	-1.46<br>[-10.37, 7.27]		-0.07<br>[-0.51, 0.36]		4.30<br>[-5.47, 13.05]	10.58<br>[1.17, 19.30]	0.21<br>[-0.27, 0.64]	0.52<br>[0.06, 0.95]
WHOQOL-BREF (environmental)	3.61<br>[-0.15, 7.18]	1.78<br>[-2.12, 5.57]	0.28<br>[-0.01, 0.56]	0.14<br>[-0.16, 0.43]	5.63<br>[-0.45, 12.22]		0.44<br>[-0.03, 0.95]		-2.75<br>[-7.59, 2.60]	-0.42<br>[-5.71, 5.37]	-0.21<br>[-0.59, 0.20]	-0.03<br>[-0.44, 0.42]
WSAS	-1.53<br>[-3.99, 1.03]	0.27<br>[-2.19, 2.97]	-0.22<br>[-0.56, 0.15]	0.04<br>[-0.31, 0.42]	-0.48<br>[-4.16, 3.17]		-0.07<br>[-0.59, 0.45]		-0.47<br>[-4.17, 2.88]	-3.38<br>[-7.23, 0.12]	-0.07<br>[-0.59, 0.41]	-0.48<br>[-1.02, 0.02]
RSQ-10D (brooding)	-0.27<br>[-1.06, 0.58]	-0.42<br>[-1.27, 0.44]	-0.09<br>[-0.37, 0.20]	-0.15<br>[-0.45, 0.15]	-0.08<br>[-1.18, 1.12]	-0.28<br>[-1.35, 0.80]	-0.03<br>[-0.41, 0.39]	-0.10<br>[-0.47, 0.28]	-0.83<br>[-1.92, 0.26]	-0.80<br>[-2.04, 0.42]	-0.29<br>[-0.67, 0.09]	-0.28<br>[-0.71, 0.15]
RSQ-10D (reflection)	0.06<br>[-0.85, 1.05]	-0.68<br>[-1.72, 0.24]	0.02<br>[-0.28, 0.35]	-0.23<br>[-0.57, 0.08]	0.10<br>[-1.21, 1.32]	-0.82<br>[-1.97, 0.45]	0.03<br>[-0.40, 0.44]	-0.27<br>[-0.66, 0.15]	-1.54<br>[-2.82, -0.28]	-0.58<br>[-1.90, 0.77]	-0.51<br>[-0.94, -0.09]	-0.19<br>[-0.63, 0.26]

Note. HSCL-11 = Hopkins Symptom Checklist 11; RSQ-10D = Response Style Questionnaire (10-item version); PTQ = Perseverative Thinking Questionnaire; DERS-16 = Difficulties in Emotion Regulation Questionnaire (16-item version); CERQ (adaptive) = Cognitive Emotion Regulation Questionnaire (adaptive subscale); CERQ (maladaptive) = Cognitive Emotion Regulation Questionnaire (maladaptive subscale); PASAT = Paced Auditory Serial Addition Test; PHQ-9 = Patient Health Questionnaire 9; GAD-7 = Generalized Anxiety Disorder Scale 7; mini-SPIN = Mini-form of the Social Phobia Inventory; WHOQOL-BREF (physical) = World Health Organization Quality of Life Brief Form; WHOQOL-BREF (psychological) = World Health Organization Quality of Life Brief Form; WHOQOL-BREF (social) = World Health Organization Quality of Life Brief Form; WHOQOL-BREF (environmental) = World Health Organization Quality of Life Brief Form; WSAS = Work and Social Adjustment Scale; RSQ-10D (brooding) = Response Style Questionnaire (brooding subfacet); RSQ-10D (reflection) = Response Style Questionnaire (reflection subfacet)

Table S4

Means and Standard Deviations at Baseline, End-of-training, and 3-month and 6-month Follow-Up

Outcome		Baseline		End-of-Training		Three-Month Follow-Up		Six-Month Follow-Up	
		aPASAT	SOR	aPASAT	SOR	aPASAT	SOR	aPASAT	SOR
	n	2	1	2	1	2	1	1	1
PTSD-7	M	23.00	24.00	21.50	21.00	17.00	18.00	13.00	19.00
	SD	2.83		0.71		7.07			
	n	0	3	0	3	0	3	0	3
PHQ-15	M		10.00		9.33		10.33		11.00
	SD		4.36		4.93		6.81		7.21
	n	2	0	2	0	1	0	2	0
EDE-Q8	M	4.12		3.50		3.75		4.56	
	SD	0.53		0.17				0.26	
	n	0	0	0	0	0	0	0	0
ANDM-20	M								
	SD								
	n	2	1	1	1	1	1	1	1
MaCS	M	12.00	1.00	9.00	1.00	0.01	1.00	0.01	1.00
	SD	1.41							
	n	2	2	2	2	2	1	1	1
BSL-23	M	44.50	42.50	38.00	29.50	37.00	19.00	30.00	19.00
	SD	0.71	2.12	4.24	7.78	16.97			
	n	2	0	2	0	1	0	2	0
OCI-R	M	1.52		1.69		0.67		1.61	
	SD	1.14		1.29				1.41	
	n	40	40	0	0	28	28	25	29
WHOQOL-BREF (physical)	M	54.91	62.05			62.25	68.37	62.29	66.50
	SD	18.91	14.97			16.19	14.44	18.33	12.93
	n	40	40	0	0	28	28	25	29
WHOQOL-BREF (psychological)	M	44.58	48.02			49.11	51.94	45.17	53.88
	SD	18.97	18.63			17.84	17.29	19.34	17.88
	n	40	40	0	0	28	28	25	29
WHOQOL-BREF (social)	M	55.83	54.37			54.17	58.63	44.67	57.18
	SD	22.42	19.97			22.85	20.35	21.23	20.26
	n	40	40	0	0	28	28	25	29
WHOQOL-BREF (environmental)	M	66.80	72.42			70.54	74.00	69.12	74.35
	SD	18.13	13.12			16.44	12.84	14.83	10.80
	n	40	40	0	0	28	28	25	29
WSAS	M	19.92	19.45			18.93	17.04	20.52	16.66
	SD	8.74	8.89			8.81	7.10	10.72	7.74

Note. PTSD-7 = Short Screening Scale for PTSD; PHQ-15 = Patient Health Questionnaire 15; EDE-Q8 = Eating Disorder Examination Questionnaire 8; ANDM-20 = Adjustment Disorder – New Module; MaCS = Mannheimer Craving Scale; BSL-23 = Borderline Symptom List 23; OCI-R = Obsessive-Compulsive Inventory-Revised; WHOQOL-BREF (physical) = World Health Organization Quality of Life Brief Form; WHOQOL-BREF (psychological) = World Health Organization Quality of Life Brief Form; WHOQOL-BREF (social) = World Health Organization Quality of Life Brief Form; WHOQOL-BREF (environmental) = World Health Organization Quality of Life Brief Form; WSAS = Work and Social Adjustment Scale

Table S5

Number of Measurements and Patients in the Random Intercept Models (ITT Analysis)

Outcome	Number of measurements	Number of patients
HSCL-11	261	80
RSQ-10D	260	80
PTQ	260	80
DERS-16	260	80
CERQ (adaptive)	260	80
CERQ (maladaptive)	260	80
PASAT	241	80
PHQ-9	211	65
GAD-7	102	31
mini-SPIN	70	21
WHOQOL-BREF (physical)	190	80
WHOQOL-BREF (psychological)	190	80
WHOQOL-BREF (social)	190	80
WHOQOL-BREF (environmental)	190	80
WSAS	190	80
RSQ-10D (brooding)	260	80
RSQ-10D (reflection)	260	80

Note. HSCL-11 = Hopkins Symptom Checklist 11; RSQ-10D = Response Style Questionnaire (10-item version); PTQ = Perseverative Thinking Questionnaire; DERS-16 = Difficulties in Emotion Regulation Questionnaire (16-item version); CERQ (adaptive) = Cognitive Emotion Regulation Questionnaire (adaptive subscale); CERQ (maladaptive) = Cognitive Emotion Regulation Questionnaire (maladaptive subscale); PASAT = Paced Auditory Serial Addition Test; PHQ-9 = Patient Health Questionnaire 9; GAD-7 = Generalized Anxiety Disorder Scale 7; mini-SPIN = Mini-form of the Social Phobia Inventory; WHOQOL-BREF (physical) = World Health Organization Quality of Life Brief Form; WHOQOL-BREF (psychological) = World Health Organization Quality of Life Brief Form; WHOQOL-BREF (social) = World Health Organization Quality of Life Brief Form; WHOQOL-BREF (environmental) = World Health Organization Quality of Life Brief Form; WSAS = Work and Social Adjustment Scale; RSQ-10D (brooding) = Response Style Questionnaire (brooding subfacet); RSQ-10D (reflection) = Response Style Questionnaire (reflection subfacet)

Table S6

Effects of Complete Case Status on Outcomes: Comparison of the Standard Model and a Pattern Mixture Model

Outcome	df	LR	p
HSCL-11	4	3.76	.440
RSQ-10D	4	2.60	.627
PTQ	4	3.96	.411
DERS-16	4	2.00	.735
CERQ (adaptive)	4	3.56	.469
CERQ (maladaptive)	4	5.11	.276
PASAT	4	3.76	.440

Note. LR = likelihood ratio; HSCL-11 = Hopkins Symptom Checklist 11; RSQ-10D = Response Style Questionnaire (10-item version); PTQ = Perseverative Thinking Questionnaire; DERS-16 = Difficulties in Emotion Regulation Questionnaire (16-item version); CERQ (adaptive) = Cognitive Emotion Regulation Questionnaire (adaptive subscale); CERQ (maladaptive) = Cognitive Emotion Regulation Questionnaire (maladaptive subscale); PASAT = Paced Auditory Serial Addition Test

Table S7

Estimates and Effect Sizes for Direct Comparisons Between Speed of Response and Adaptive Paced Serial Addition Test Training with 95% Confidence Interval

Outcome	Time (end-of-training)		Training x Time (end-of-training)		Time (3-month follow-up)		Training x Time (3-month follow-up)		Time (6-month follow-up)		Training x Time (6-month follow-up)	
	B	*d*	B	*d*	B	*d*	B	*d*	B	*d*	B	*d*
HSCL-11	-0.14<br>[-0.29, 0.01]	-0.17<br>[-0.32, -0.01]	-0.24<br>[-0.50, 0.02]	-0.29<br>[-0.55, -0.01]	-0.18<br>[-0.42, 0.08]	-0.01<br>[-0.19, 0.19]	-0.30<br>[-0.72, 0.14]	-0.01<br>[-0.32, 0.32]	0.02<br>[-0.17, 0.23]	0.01<br>[-0.20, 0.22]	0.04<br>[-0.30, 0.40]	0.01<br>[-0.34, 0.38]
RSQ-10D	-0.49<br>[-2.13, 1.03]	-0.94<br>[-2.59, 0.64]	-0.09<br>[-0.41, 0.20]	-0.18<br>[-0.50, 0.12]	-0.47<br>[-2.89, 1.88]	-1.20<br>[-3.15, 0.74]	-0.09<br>[-0.55, 0.36]	-0.23<br>[-0.60, 0.14]	-2.27<br>[-4.31, -0.27]	-1.88<br>[-4.10, 0.32]	-0.44<br>[-0.83, -0.05]	-0.36<br>[-0.79, 0.06]
PTQ	-4.09<br>[-7.22, -0.72]	-4.71<br>[-8.06, -1.29]	-0.35<br>[-0.62, -0.06]	-0.40<br>[-0.69, -0.11]	-5.06<br>[-10.10, -0.26]	-2.73<br>[-6.62, 1.41]	-0.43<br>[-0.86, -0.02]	-0.23<br>[-0.57, 0.12]	-0.53<br>[-4.69, 3.93]	0.41<br>[-3.77, 4.92]	-0.05<br>[-0.40, 0.34]	0.03<br>[-0.32, 0.42]
DERS-16	-5.65<br>[-8.91, -2.50]	-5.31<br>[-8.65, -2.19]	-0.39<br>[-0.62, -0.17]	-0.37<br>[-0.60, -0.15]	-7.98<br>[-14.25, -1.90]	4.40<br>[0.63, 8.38]	-0.56<br>[-0.99, -0.13]	0.31<br>[0.04, 0.58]	4.00<br>[-0.50, 8.22]	2.59<br>[-1.88, 6.88]	0.28<br>[-0.03, 0.57]	0.18<br>[-0.13, 0.48]
CERQ (adaptive)	0.65<br>[-2.65, 3.76]	2.21<br>[-1.28, 5.70]	0.05<br>[-0.20, 0.28]	0.17<br>[-0.10, 0.43]	4.90<br>[-0.39, 10.14]	-0.36<br>[-4.39, 3.72]	0.37<br>[-0.03, 0.77]	-0.03<br>[-0.33, 0.28]	-1.95<br>[-6.42, 2.34]	-4.42<br>[-9.00, 0.11]	-0.15<br>[-0.48, 0.18]	-0.33<br>[-0.68, 0.01]
CERQ (maladaptive)	-2.67<br>[-5.22, 0.04]	-2.42<br>[-4.91, 0.59]	-0.25<br>[-0.49, 0.00]	-0.23<br>[-0.47, 0.06]	-2.60<br>[-7.32, 2.36]	0.25<br>[-3.21, 3.98]	-0.25<br>[-0.69, 0.22]	0.02<br>[-0.30, 0.38]	-0.15<br>[-3.93, 3.58]	-0.09<br>[-4.04, 3.36]	-0.01<br>[-0.37, 0.34]	-0.01<br>[-0.38, 0.32]
PASAT	0.69<br>[0.68, 0.72]	0.77<br>[0.76, 0.79]	1.73<br>[1.69, 1.79]	1.92<br>[1.89, 1.95]	-0.03<br>[0.00, 0.08]	-0.61<br>[-0.62, -0.58]	-0.07<br>[0.01, 0.21]	-1.51<br>[-1.55, -1.43]	-0.43<br>[-0.46, -0.38]	-0.47<br>[-0.51, -0.43]	-1.07<br>[-1.15, -0.95]	-1.16<br>[-1.26, -1.06]
PHQ-9	-2.10<br>[-3.67, -0.60]	-2.11<br>[-3.74, -0.49]	-0.47<br>[-0.82, -0.13]	-0.47<br>[-0.83, -0.11]	-2.28<br>[-4.59, -0.19]	0.37<br>[-1.67, 2.27]	-0.51<br>[-1.02, -0.04]	0.08<br>[-0.37, 0.50]	0.83<br>[-1.06, 2.80]	0.64<br>[-1.55, 2.82]	0.19<br>[-0.24, 0.62]	0.14<br>[-0.35, 0.63]
GAD-7	-2.94<br>[-4.83, -1.09]	-0.98<br>[-2.84, 0.87]	-0.70<br>[-1.15, -0.26]	-0.23<br>[-0.67, 0.21]	-1.97<br>[-5.45, 1.45]	0.04<br>[-2.13, 2.28]	-0.47<br>[-1.29, 0.34]	0.01<br>[-0.50, 0.54]	1.69<br>[-0.75, 4.10]	0.81<br>[-1.63, 3.22]	0.40<br>[-0.18, 0.97]	0.19<br>[-0.39, 0.76]
mini-SPIN	-1.43<br>[-2.61, -0.21]	-0.80<br>[-2.13, 0.53]	-0.44<br>[-0.81, -0.07]	-0.25<br>[-0.66, 0.17]	-4.06<br>[-6.28, -1.66]	-0.03<br>[-1.69, 1.50]	-1.26<br>[-1.95, -0.51]	-0.01<br>[-0.52, 0.47]	1.88<br>[0.18, 3.50]	2.01<br>[-0.07, 3.80]	0.58<br>[0.06, 1.08]	0.62<br>[-0.02, 1.18]
WHOQOL-BREF (physical)	8.76<br>[4.27, 13.86]	9.24<br>[4.17, 14.89]	0.61<br>[0.30, 0.96]	0.64<br>[0.29, 1.03]	10.71<br>[2.59, 18.80]		0.74<br>[0.18, 1.30]		-3.84<br>[-10.96, 3.12]	-5.34<br>[-12.58, 1.33]	-0.27<br>[-0.76, 0.22]	-0.37<br>[-0.87, 0.09]
WHOQOL-BREF (psychological)	5.04<br>[0.67, 9.81]	1.54<br>[-2.98, 6.29]	0.29<br>[0.04, 0.57]	0.09<br>[-0.17, 0.36]	6.23<br>[-1.30, 14.71]		0.36<br>[-0.08, 0.85]		-3.66<br>[-9.73, 2.67]	3.65<br>[-2.96, 9.79]	-0.21<br>[-0.56, 0.15]	0.21<br>[-0.17, 0.57]
WHOQOL-BREF (social)	-2.69<br>[-9.77, 4.18]	-8.56<br>[-16.15, -1.18]	-0.13<br>[-0.48, 0.21]	-0.42<br>[-0.79, -0.06]	-1.20<br>[-11.07, 9.49]		-0.06<br>[-0.54, 0.47]		5.46<br>[-3.40, 14.94]	10.89<br>[0.77, 20.05]	0.27<br>[-0.17, 0.73]	0.54<br>[0.04, 0.99]
WHOQOL-BREF (environmental)	2.82<br>[-0.77, 6.45]	2.04<br>[-1.93, 5.94]	0.22<br>[-0.06, 0.50]	0.16<br>[-0.15, 0.46]	7.97<br>[1.35, 14.98]		0.62<br>[0.11, 1.17]		-2.51<br>[-7.92, 2.37]	-1.45<br>[-6.48, 3.69]	-0.20<br>[-0.62, 0.18]	-0.11<br>[-0.50, 0.29]
WSAS	-1.40<br>[-3.90, 1.08]	0.53<br>[-2.03, 3.25]	-0.20<br>[-0.55, 0.15]	0.07<br>[-0.29, 0.46]	-2.31<br>[-6.35, 1.66]		-0.32<br>[-0.89, 0.23]		-0.36<br>[-3.89, 3.27]	-3.40<br>[-7.23, 0.27]	-0.05<br>[-0.55, 0.46]	-0.48<br>[-1.02, 0.04]
RSQ-10D (brooding)	-0.51<br>[-1.32, 0.33]	-0.48<br>[-1.41, 0.42]	-0.18<br>[-0.48, 0.12]	-0.17<br>[-0.51, 0.15]	-0.34<br>[-1.63, 0.92]	-0.18<br>[-1.23, 0.92]	-0.12<br>[-0.59, 0.33]	-0.06<br>[-0.44, 0.33]	-0.65<br>[-1.82, 0.55]	-0.86<br>[-1.99, 0.35]	-0.23<br>[-0.65, 0.20]	-0.31<br>[-0.72, 0.13]
RSQ-10D (reflection)	0.03<br>[-0.86, 0.95]	-0.44<br>[-1.39, 0.53]	0.01<br>[-0.30, 0.34]	-0.16<br>[-0.49, 0.19]	-0.13<br>[-1.46, 1.18]	-1.03<br>[-2.19, 0.19]	-0.05<br>[-0.52, 0.42]	-0.37<br>[-0.78, 0.07]	-1.62<br>[-2.82, -0.42]	-1.01<br>[-2.30, 0.24]	-0.57<br>[-1.00, -0.15]	-0.36<br>[-0.82, 0.09]

Note. HSCL-11 = Hopkins Symptom Checklist 11; RSQ-10D = Response Style Questionnaire (10-item version); PTQ = Perseverative Thinking Questionnaire; DERS-16 = Difficulties in Emotion Regulation Questionnaire (16-item version); CERQ (adaptive) = Cognitive Emotion Regulation Questionnaire (adaptive subscale); CERQ (maladaptive) = Cognitive Emotion Regulation Questionnaire (maladaptive subscale); PASAT = Paced Auditory Serial Addition Test; PHQ-9 = Patient Health Questionnaire 9; GAD-7 = Generalized Anxiety Disorder Scale 7; mini-SPIN = Mini-form of the Social Phobia Inventory; WHOQOL-BREF (physical) = World Health Organization Quality of Life Brief Form; WHOQOL-BREF (psychological) = World Health Organization Quality of Life Brief Form; WHOQOL-BREF (social) = World Health Organization Quality of Life Brief Form; WHOQOL-BREF (environmental) = World Health Organization Quality of Life Brief Form; WSAS = Work and Social Adjustment Scale; RSQ-10D (brooding) = Response Style Questionnaire (brooding subfacet); RSQ-10D (reflection) = Response Style Questionnaire (reflection subfacet). The estimate for the PASAT task can be interpreted as change in probability of correct response for a given trial. aPASAT training is the reference condition (effects of time). The interactions display the difference of the effect of time between training conditions from baseline to either post-training, 3- or 6-month follow-up assessment.

Table S8

Number of Measurements and Patients in the Random Intercept Models (PP Analysis)

Outcome	Number of measurements	Number of patients
HSCL-11	234	66
RSQ-10D	234	66
PTQ	234	66
DERS-16	234	66
CERQ (adaptive)	234	66
CERQ (maladaptive)	234	66
PASAT	218	66
PHQ-9	191	54
GAD-7	90	26
mini-SPIN	61	17
WHOQOL-BREF (physical)	168	66
WHOQOL-BREF (psychological)	168	66
WHOQOL-BREF (social)	168	66
WHOQOL-BREF (environmental)	168	66
WSAS	168	66
RSQ-10D (brooding)	234	66
RSQ-10D (reflection)	234	66

Note. HSCL-11 = Hopkins Symptom Checklist 11; RSQ-10D = Response Style Questionnaire (10-item version); PTQ = Perseverative Thinking Questionnaire; DERS-16 = Difficulties in Emotion Regulation Questionnaire (16-item version); CERQ (adaptive) = Cognitive Emotion Regulation Questionnaire (adaptive subscale); CERQ (maladaptive) = Cognitive Emotion Regulation Questionnaire (maladaptive subscale); PASAT = Paced Auditory Serial Addition Test; PHQ-9 = Patient Health Questionnaire 9; GAD-7 = Generalized Anxiety Disorder Scale 7; mini-SPIN = Mini-form of the Social Phobia Inventory; WHOQOL-BREF (physical) = World Health Organization Quality of Life Brief Form; WHOQOL-BREF (psychological) = World Health Organization Quality of Life Brief Form; WHOQOL-BREF (social) = World Health Organization Quality of Life Brief Form; WHOQOL-BREF (environmental) = World Health Organization Quality of Life Brief Form; WSAS = Work and Social Adjustment Scale; RSQ-10D (brooding) = Response Style Questionnaire (brooding subfacet); RSQ-10D (reflection) = Response Style Questionnaire (reflection subfacet)

Figure S3

Change in Global Mental Distress From Baseline to Follow−Up after Six Month

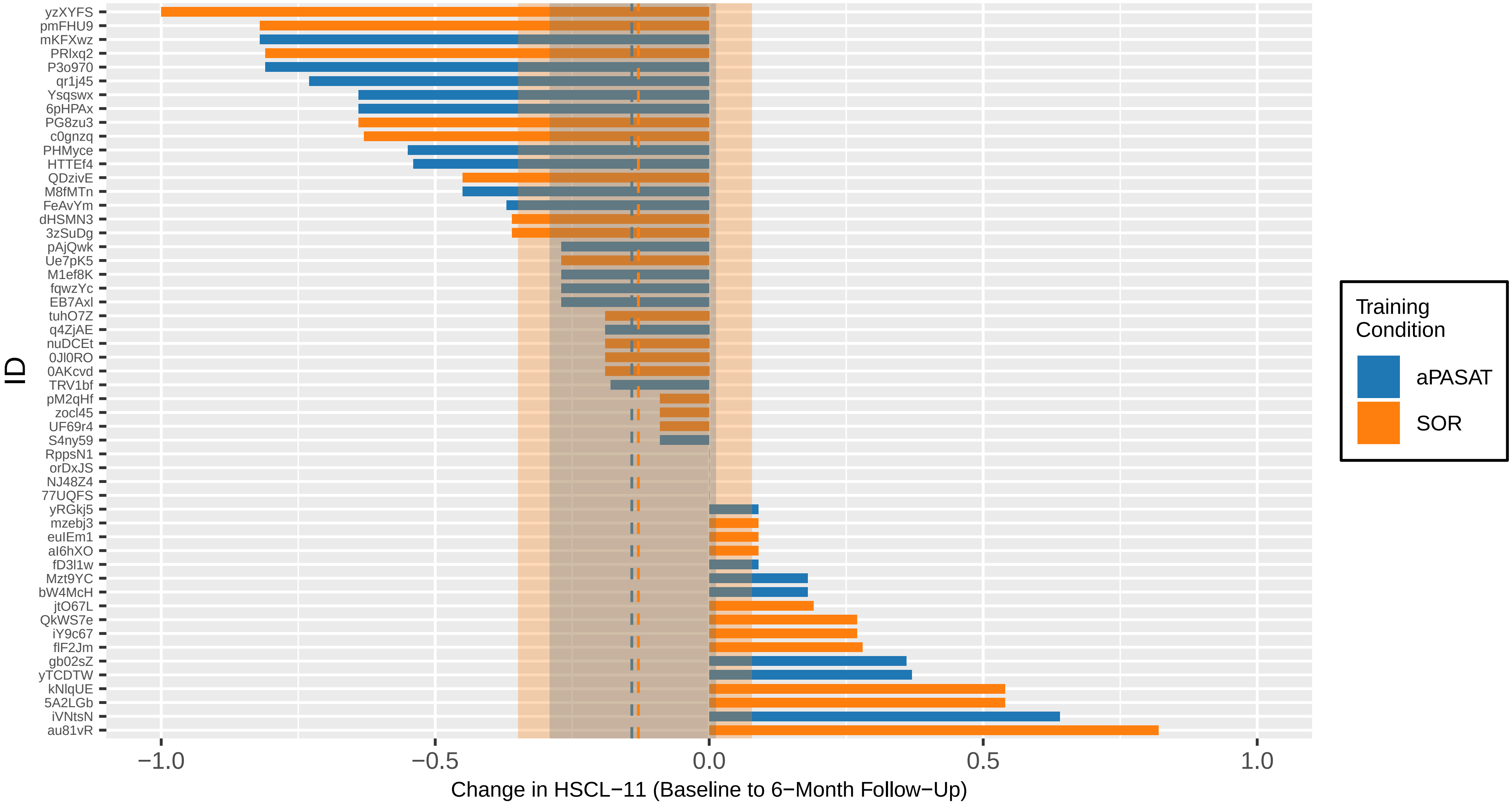

Note. HSCL−11 = Hopkins Symptom Checklist 11; Bars depict individual changes in symptom severity for each participant.
Dashed lines indicate model's predicted average reduction in symptoms for each condition, with shaded areas representing the 95% confidence interval.

Figure S4

Model−Based Change Trajectories in Rumination

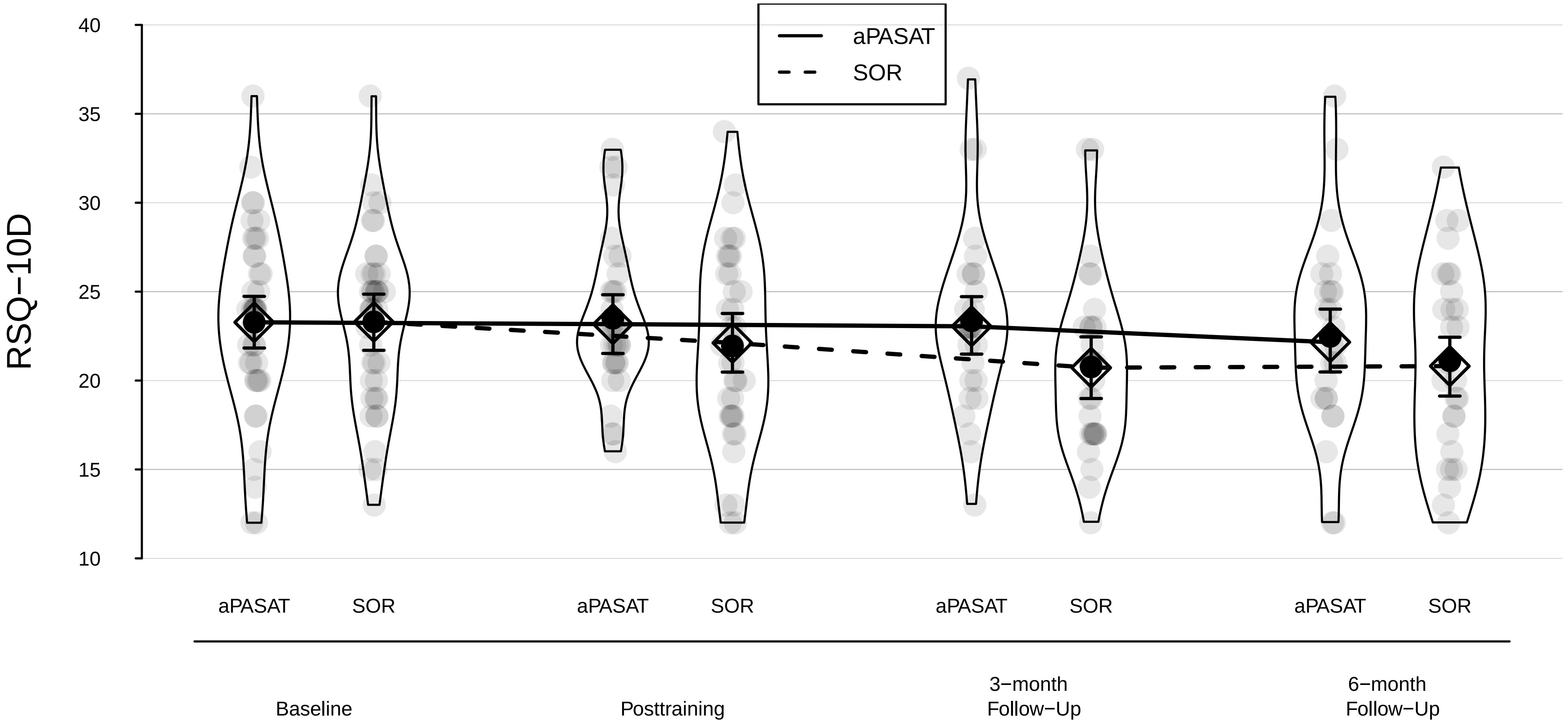

Note. RSQ−10D = Response Style Questionnaire (10−item version); Grey dots represent individual data points.
Solid black dots show empirical mean values. Diamond shapes represent model−based estimates values with 95% confidence intervals.
The width of the plot reflects the distribution of data. aPASAT = adaptive Paced Auditory Serial Addition Test Training; SOR = Speed of Response Training

Figure S5

Model−Based Change Trajectories in Repetitive Negative Thinking

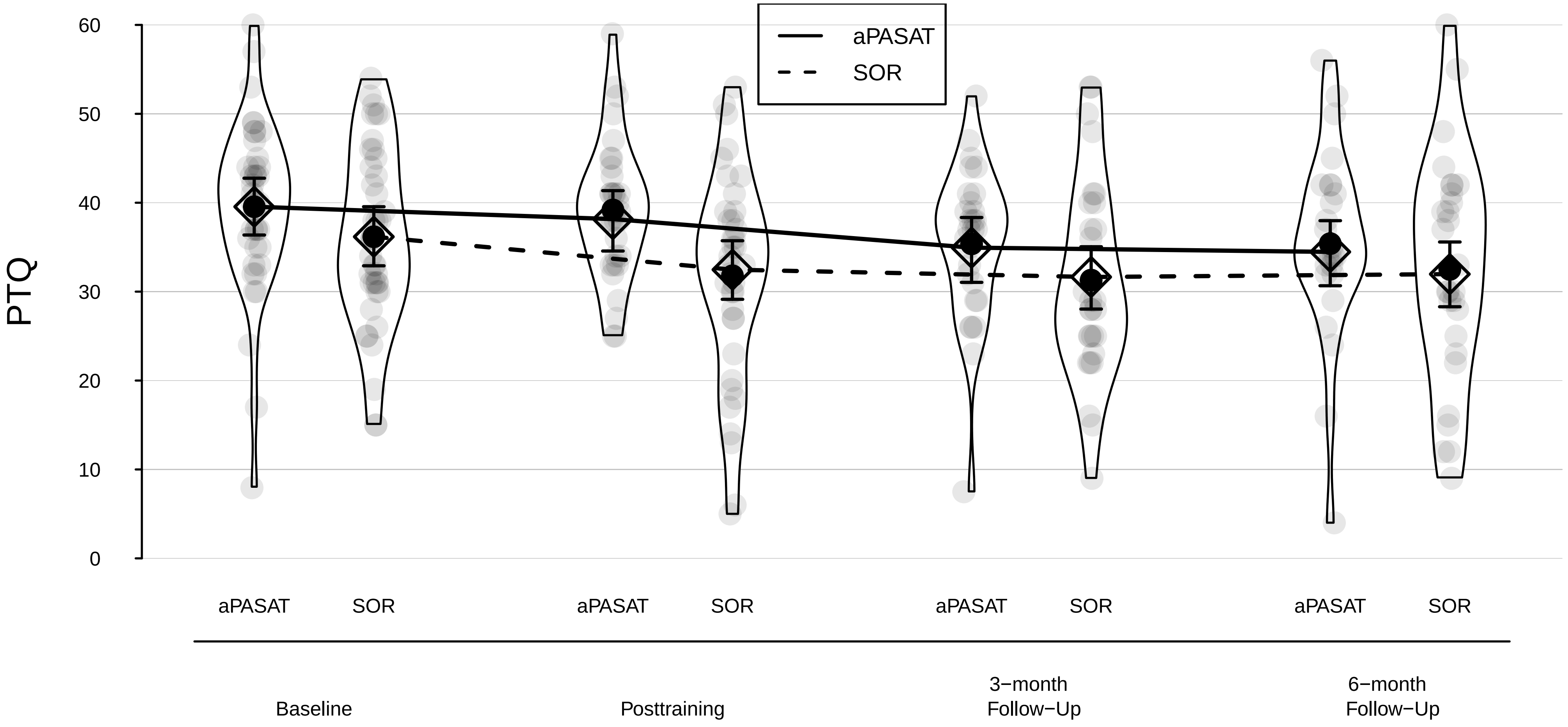

Note. PTQ = Perseverative Thinking Questionnaire; Grey dots represent individual data points.
Solid black dots show empirical mean values. Diamond shapes represent model−based estimates values with 95% confidence intervals.
The width of the plot reflects the distribution of data. aPASAT = adaptive Paced Auditory Serial Addition Test Training; SOR = Speed of Response Training

Figure S6

Model−Based Change Trajectories in Difficulties in Emotion Regulation

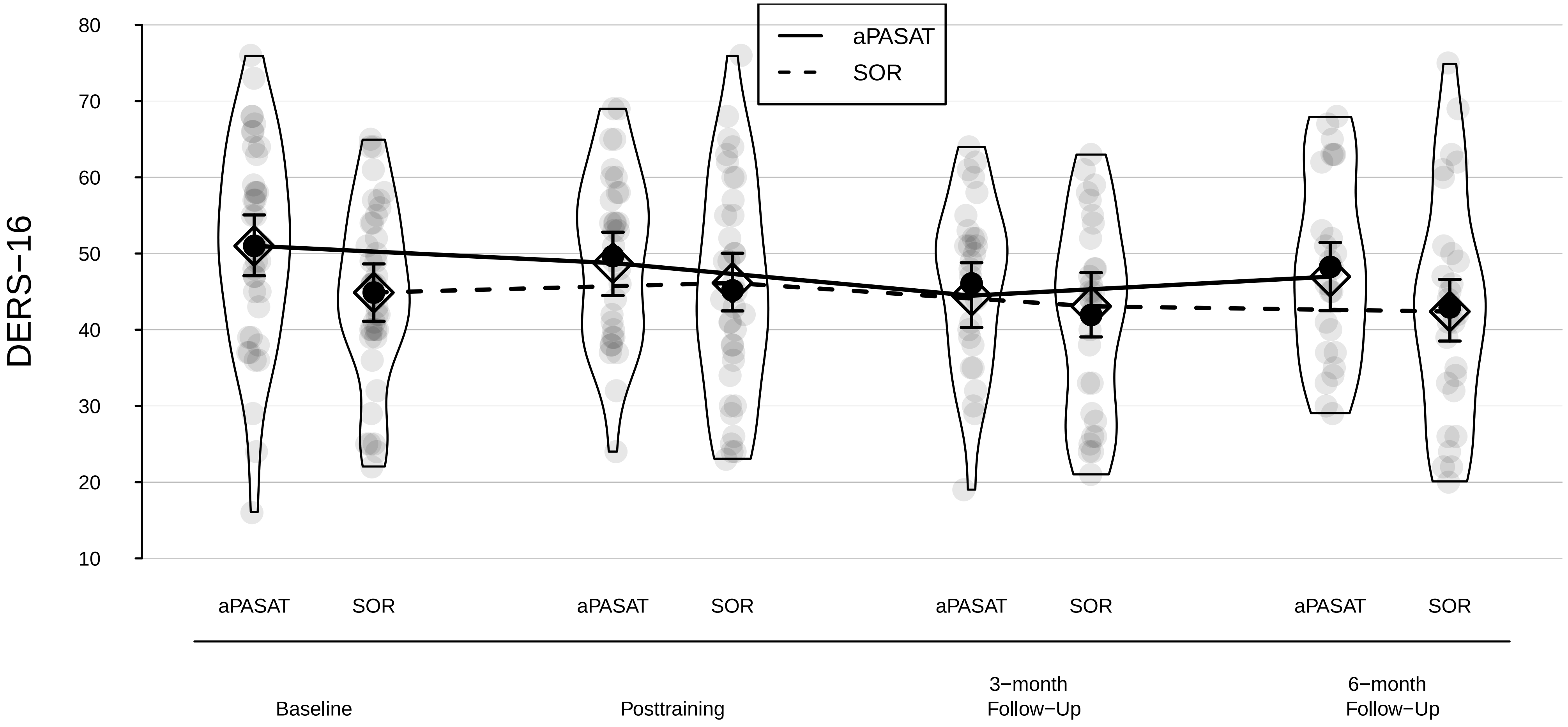

Note. DERS−16 = Difficulties in Emotion Regulation Questionnaire (16−item version); Grey dots represent individual data points.
Solid black dots show empirical mean values. Diamond shapes represent model−based estimates values with 95% confidence intervals.
The width of the plot reflects the distribution of data. aPASAT = adaptive Paced Auditory Serial Addition Test Training; SOR = Speed of Response Training

Figure S7

Model−Based Change Trajectories in Adaptive Cognitive Emotion Regulation

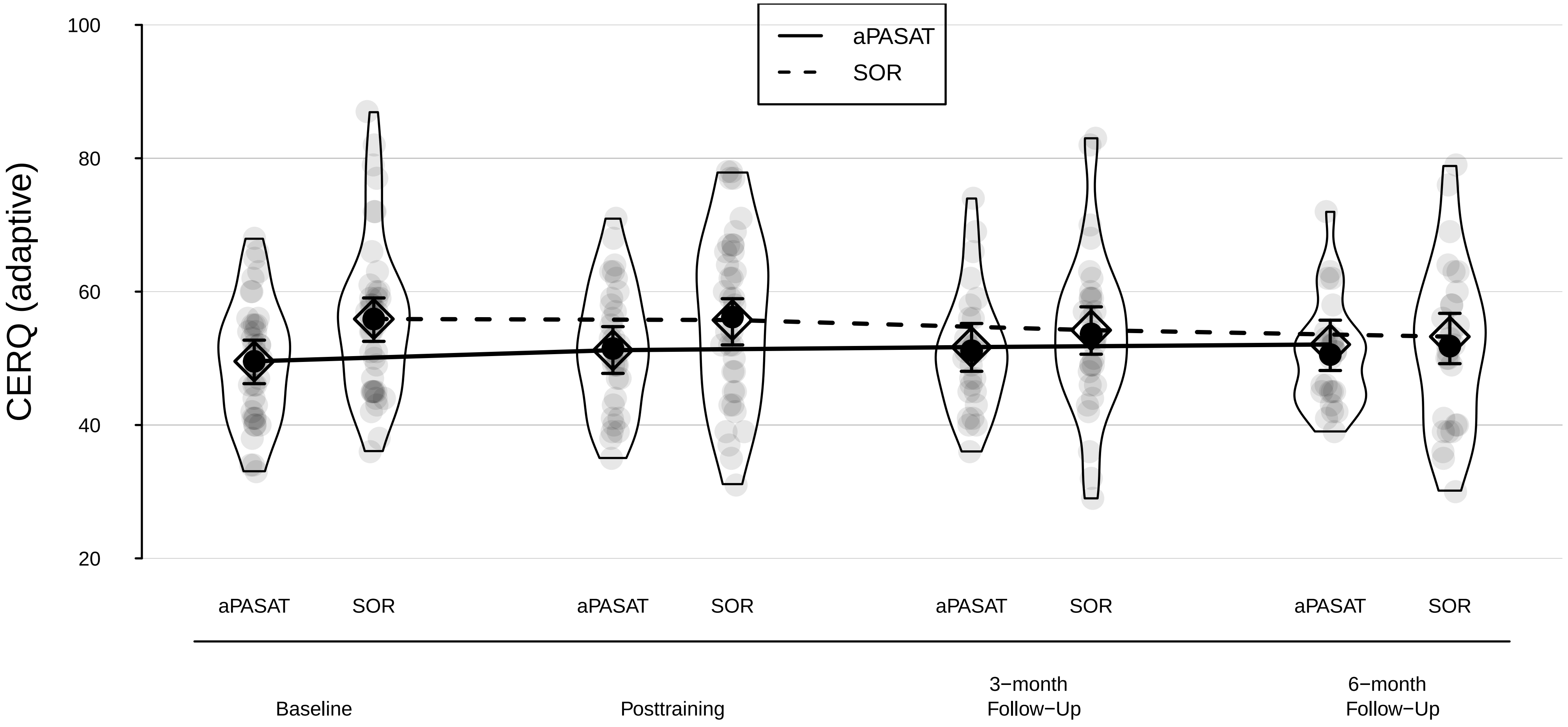

Note. CERQ (adaptive) = Cognitive Emotion Regulation Questionnaire (adaptive subscale); Grey dots represent individual data points. Solid black dots show empirical mean values. Diamond shapes represent model−based estimates values with 95% confidence intervals. The width of the plot reflects the distribution of data. aPASAT = adaptive Paced Auditory Serial Addition Test Training; SOR = Speed of Response Training

Figure S8

Model−Based Change Trajectories in Maladaptive Cognitive Emotion Regulation

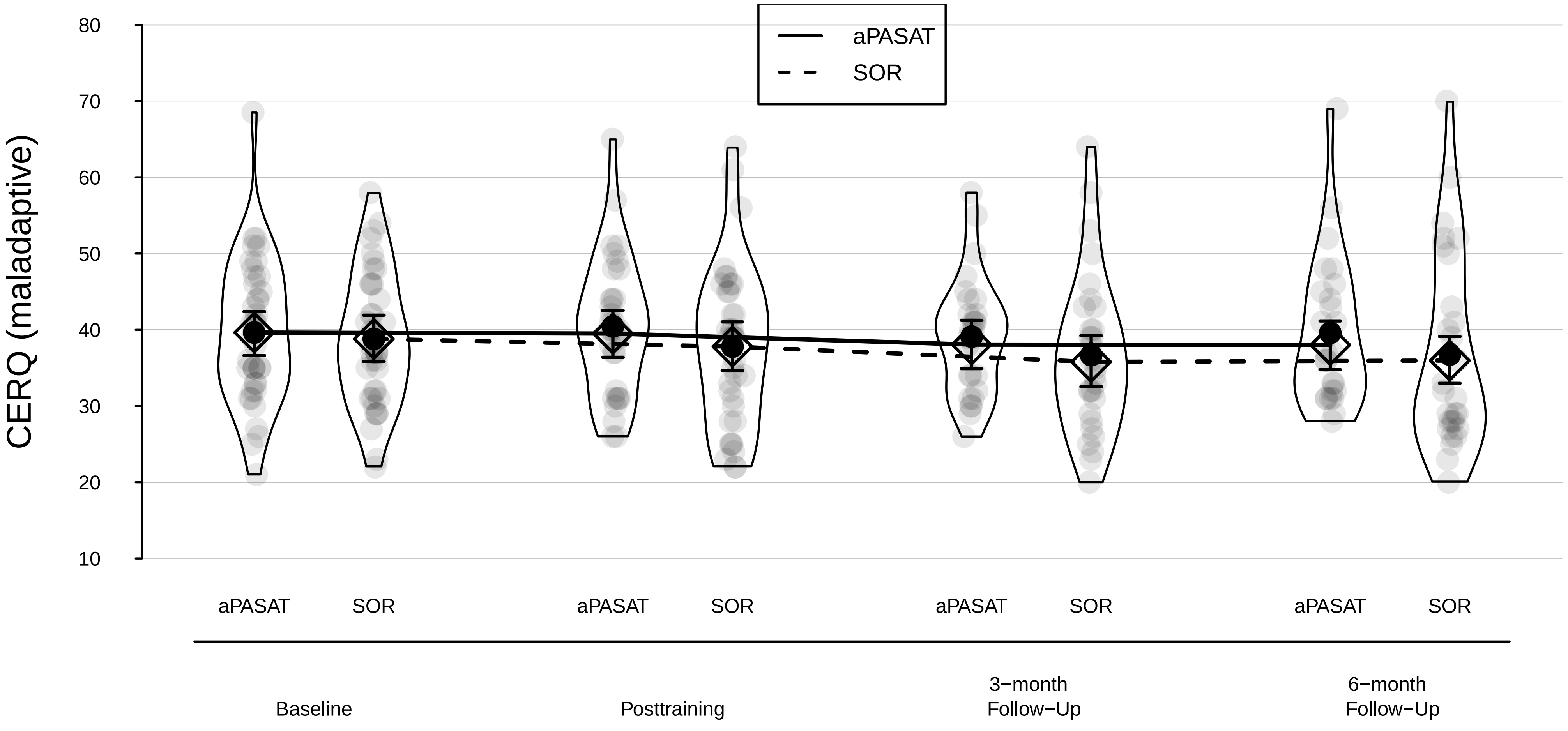

Note. CERQ (maladaptive) = Cognitive Emotion Regulation Questionnaire (maladaptive subscale); Grey dots represent individual data points. Solid black dots show empirical mean values. Diamond shapes represent model−based estimates values with 95% confidence intervals. The width of the plot reflects the distribution of data. aPASAT = adaptive Paced Auditory Serial Addition Test Training; SOR = Speed of Response Training

Figure S9

Model−Based Change Trajectories in Cognitive Control

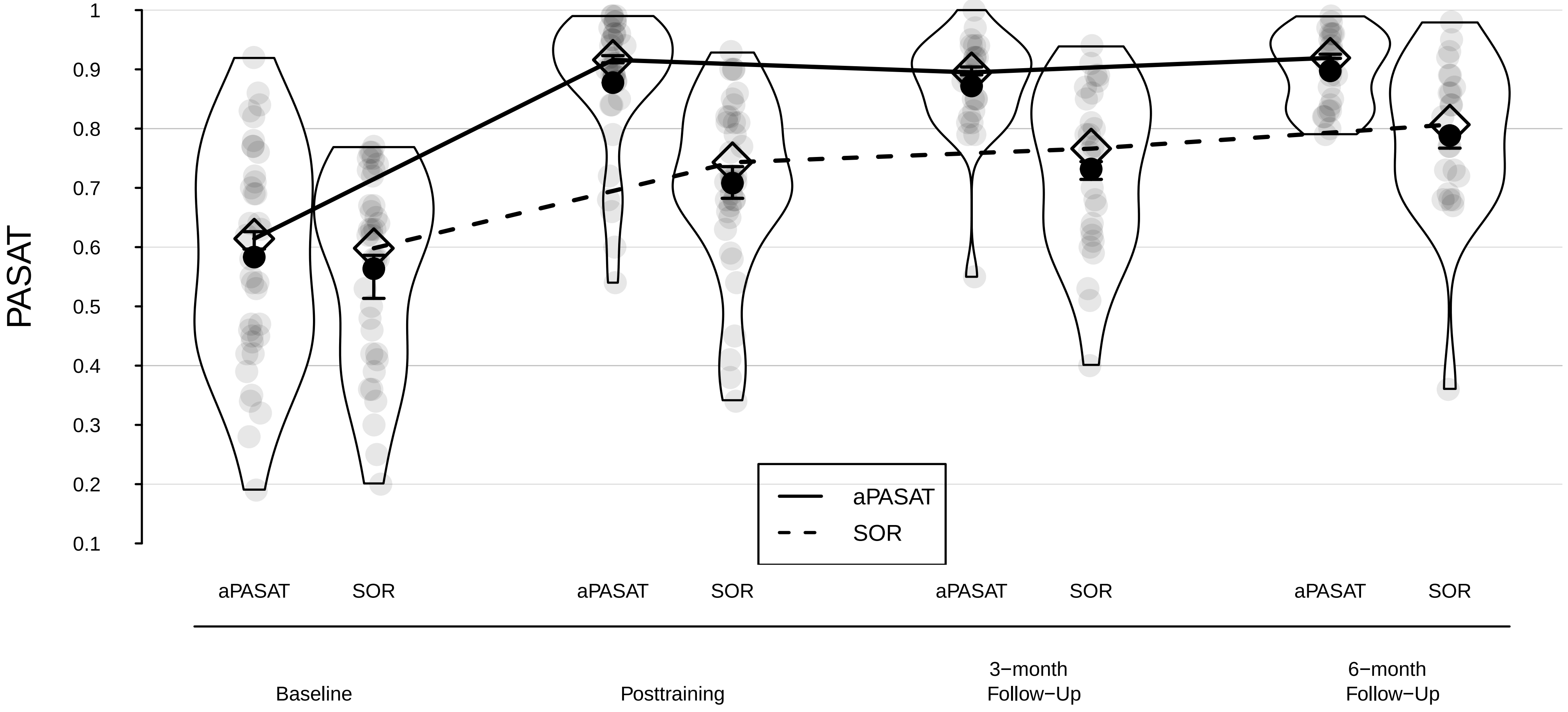

Note. PASAT = Paced Auditory Serial Addition Test; Grey dots represent individual data points.
Solid black dots show empirical mean values. Diamond shapes represent model−based estimates values with 95% confidence intervals.
The width of the plot reflects the distribution of data. aPASAT = adaptive Paced Auditory Serial Addition Test Training; SOR = Speed of Response Training

Figure S10

Model−Based Change Trajectories in Symptoms of Depression

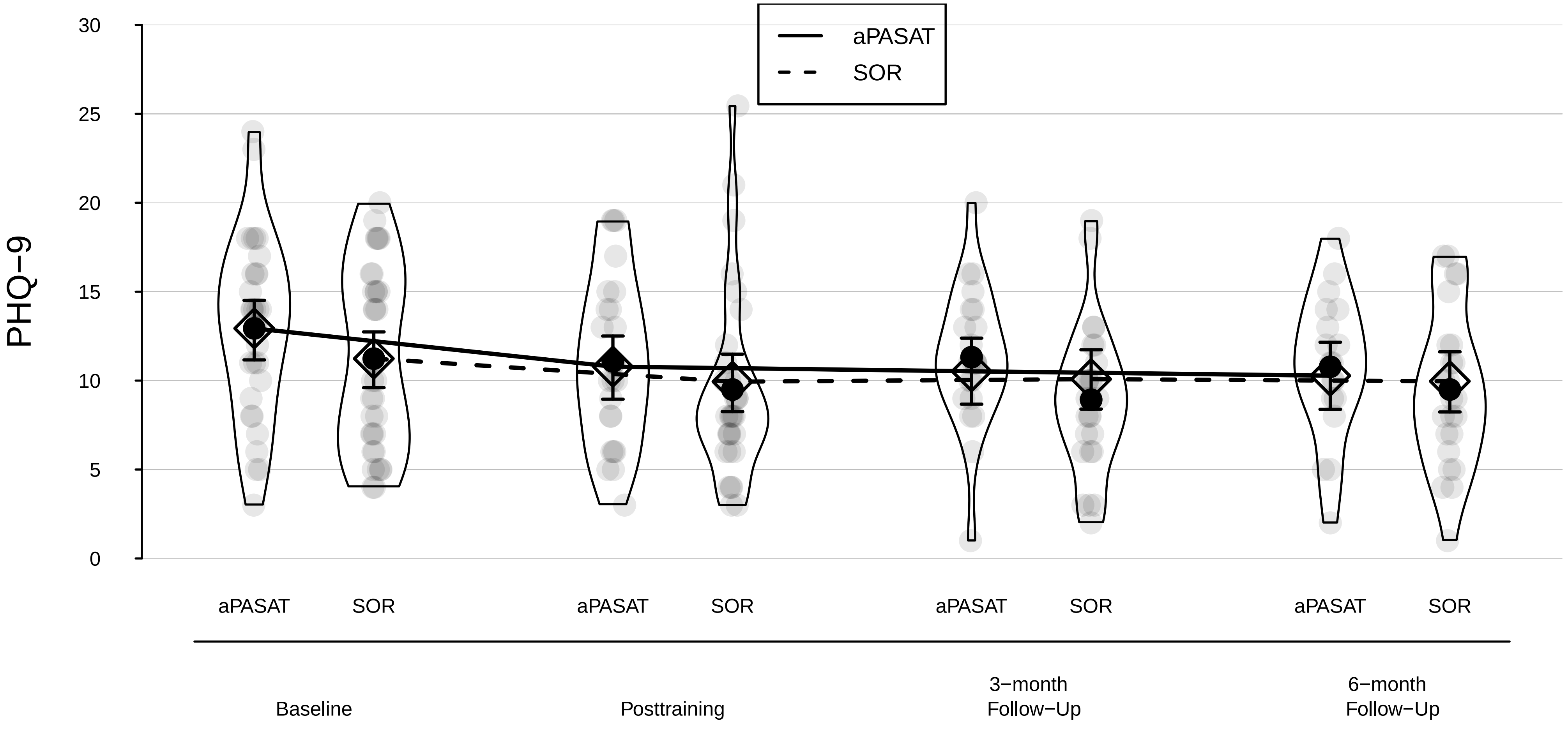

Note. PHQ−9 = Patient Health Questionnaire 9; Grey dots represent individual data points.
Solid black dots show empirical mean values. Diamond shapes represent model−based estimates values with 95% confidence intervals.
The width of the plot reflects the distribution of data. aPASAT = adaptive Paced Auditory Serial Addition Test Training; SOR = Speed of Response Training

Figure S11

Model−Based Change Trajectories in Symptoms of Anxiety

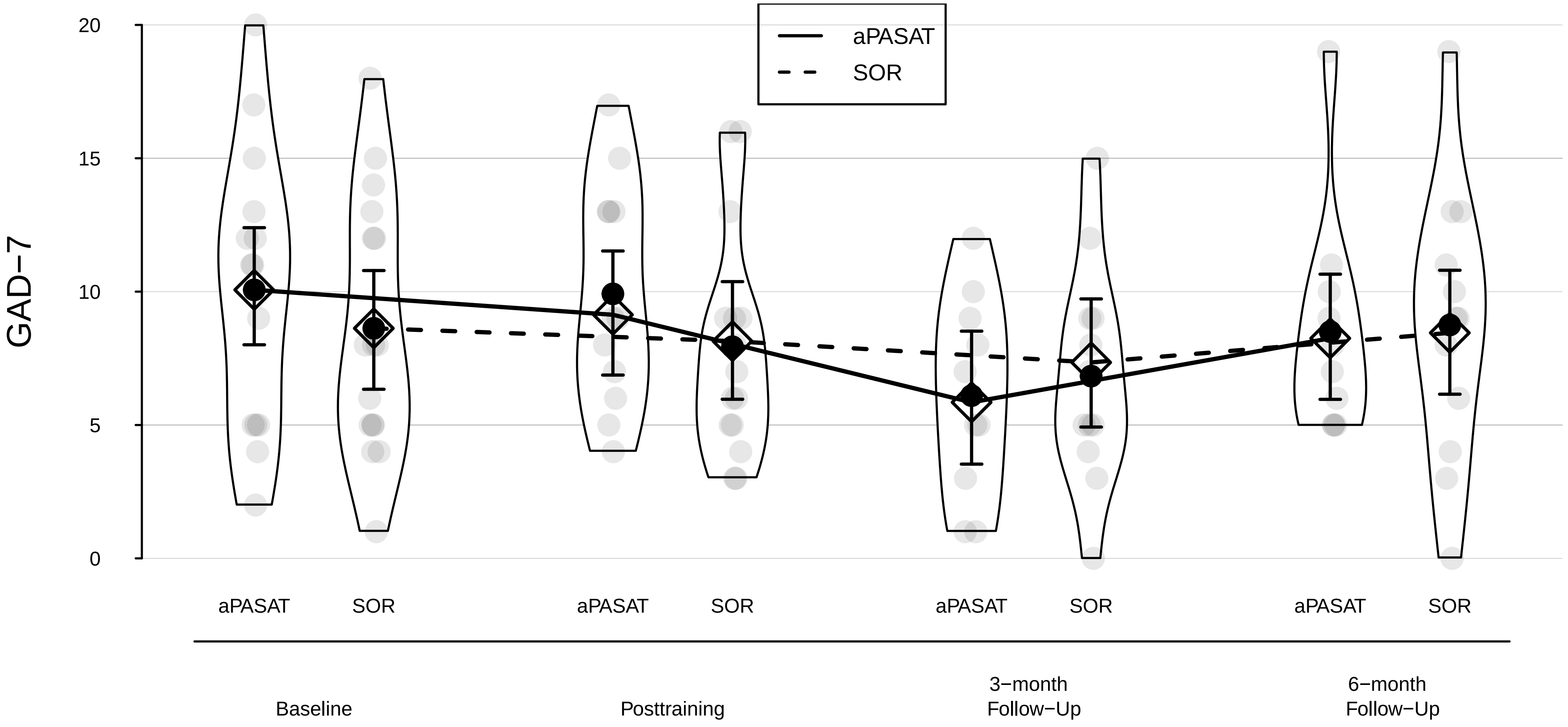

Note. GAD−7 = Generalized Anxiety Disorder Scale 7; Grey dots represent individual data points.
Solid black dots show empirical mean values. Diamond shapes represent model−based estimates values with 95% confidence intervals.
The width of the plot reflects the distribution of data. aPASAT = adaptive Paced Auditory Serial Addition Test Training; SOR = Speed of Response Training

Figure S12

Model−Based Change Trajectories in Symptoms of Social Anxiety

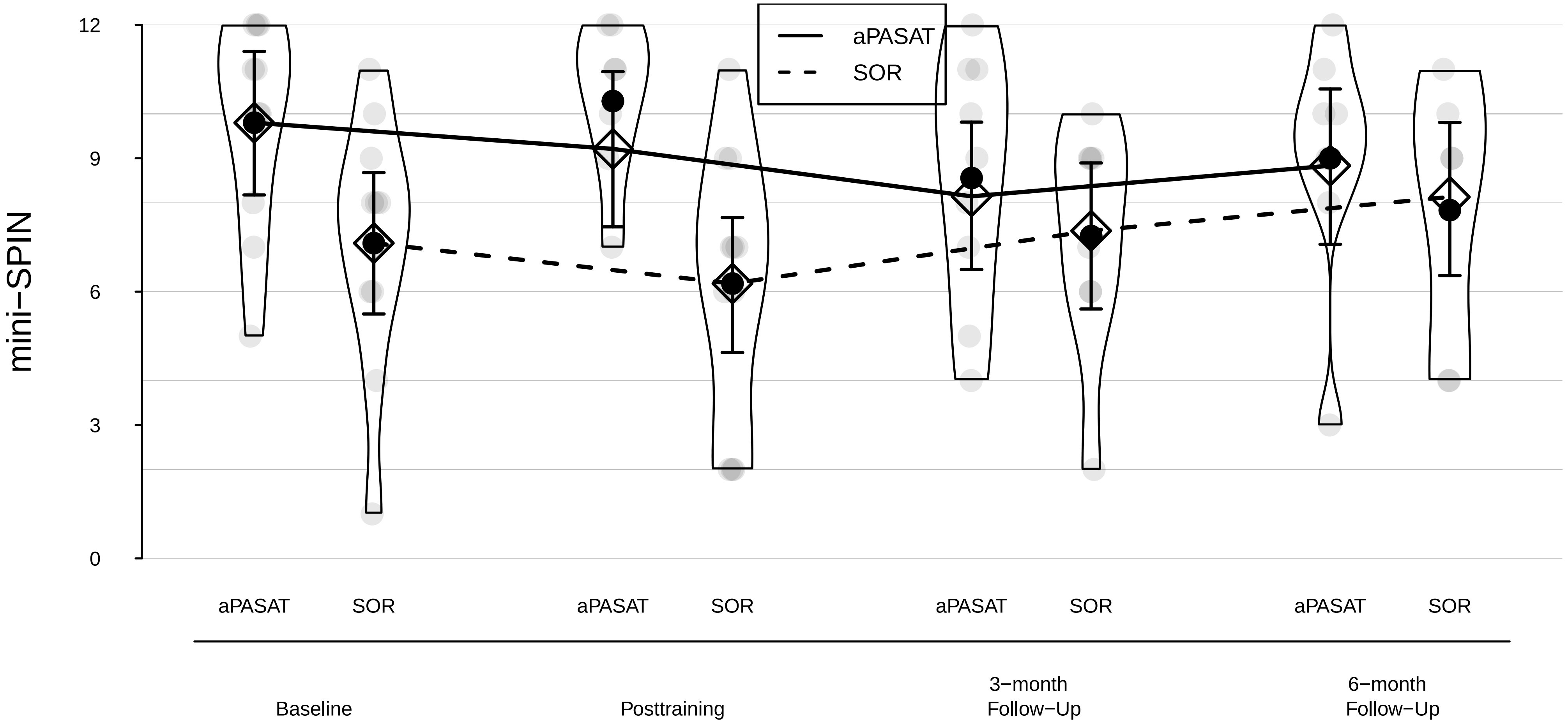

Note. mini−SPIN = Mini−form of the Social Phobia Inventory; Grey dots represent individual data points.
Solid black dots show empirical mean values. Diamond shapes represent model−based estimates values with 95% confidence intervals.
The width of the plot reflects the distribution of data. aPASAT = adaptive Paced Auditory Serial Addition Test Training; SOR = Speed of Response Training

Figure S13

Model−Based Change Trajectories in Quality of Life (physical)

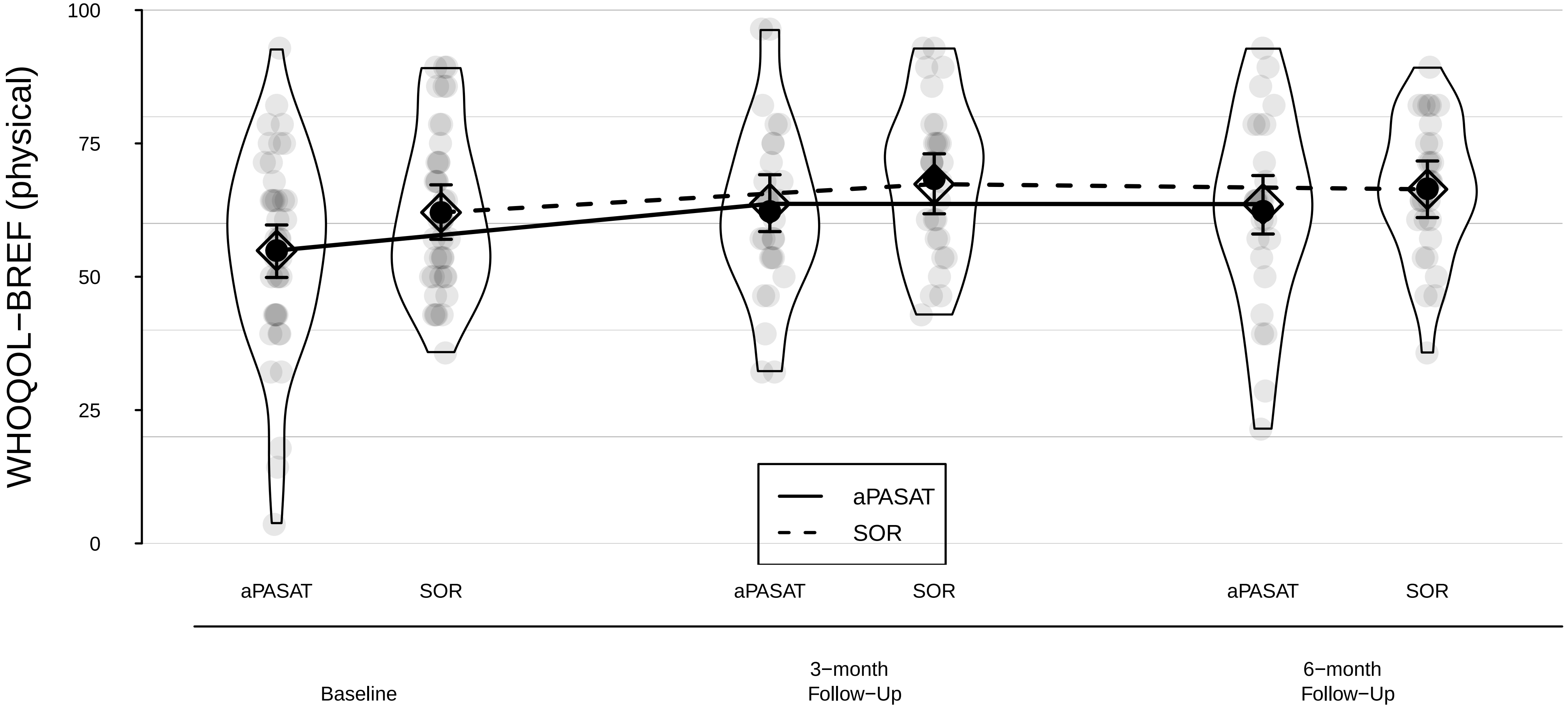

Note. WHOQOL−BREF (physical) = World Health Organization Quality of Life Brief Form; Grey dots represent individual data points.
Solid black dots show empirical mean values. Diamond shapes represent model−based estimates values with 95% confidence intervals.
The width of the plot reflects the distribution of data. aPASAT = adaptive Paced Auditory Serial Addition Test Training; SOR = Speed of Response Training

Figure S14

Model−Based Change Trajectories in Quality of Life (psychological)

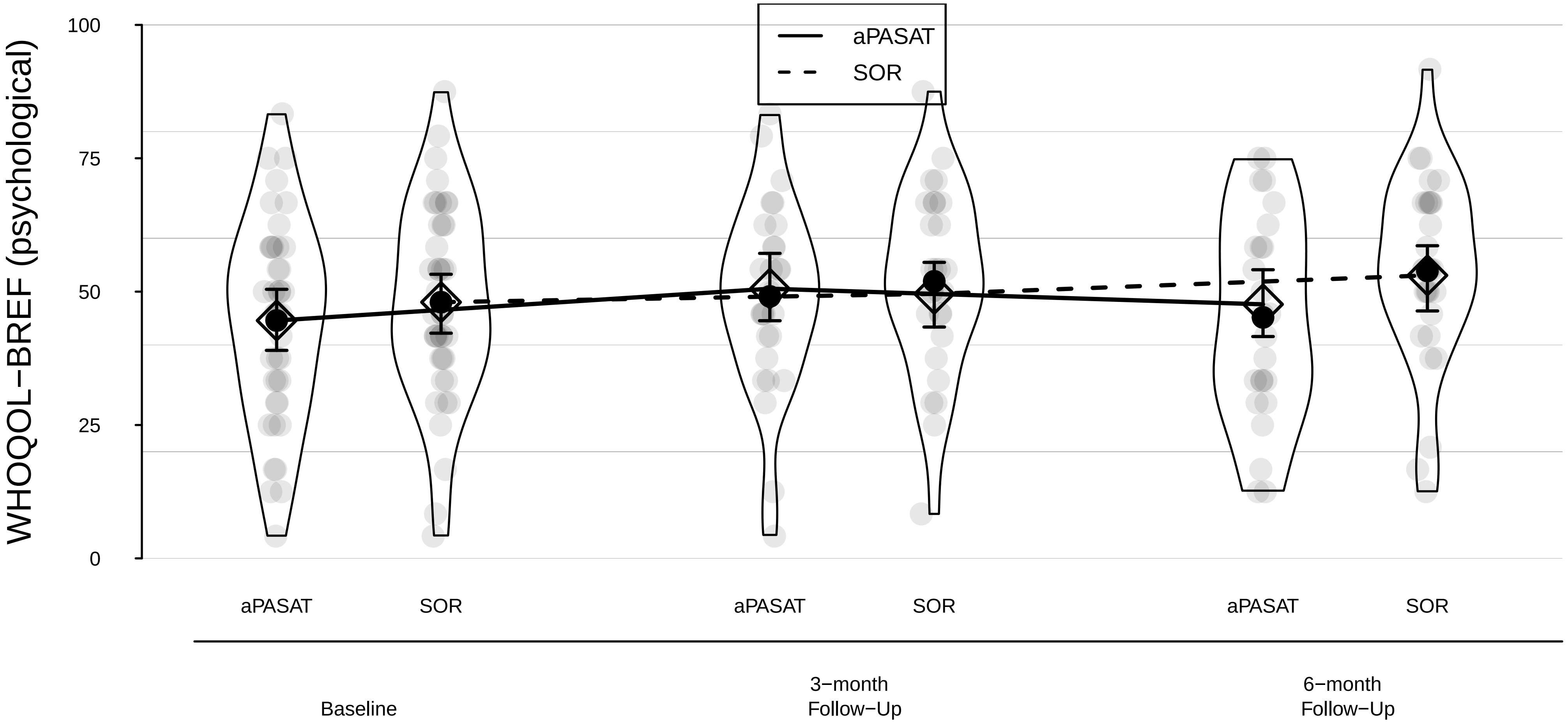

Note. WHOQOL−BREF (psychological) = World Health Organization Quality of Life Brief Form; Grey dots represent individual data points.
Solid black dots show empirical mean values. Diamond shapes represent model−based estimates values with 95% confidence intervals.
The width of the plot reflects the distribution of data. aPASAT = adaptive Paced Auditory Serial Addition Test Training; SOR = Speed of Response Training

Figure S15

Model−Based Change Trajectories in Quality of Life (social)

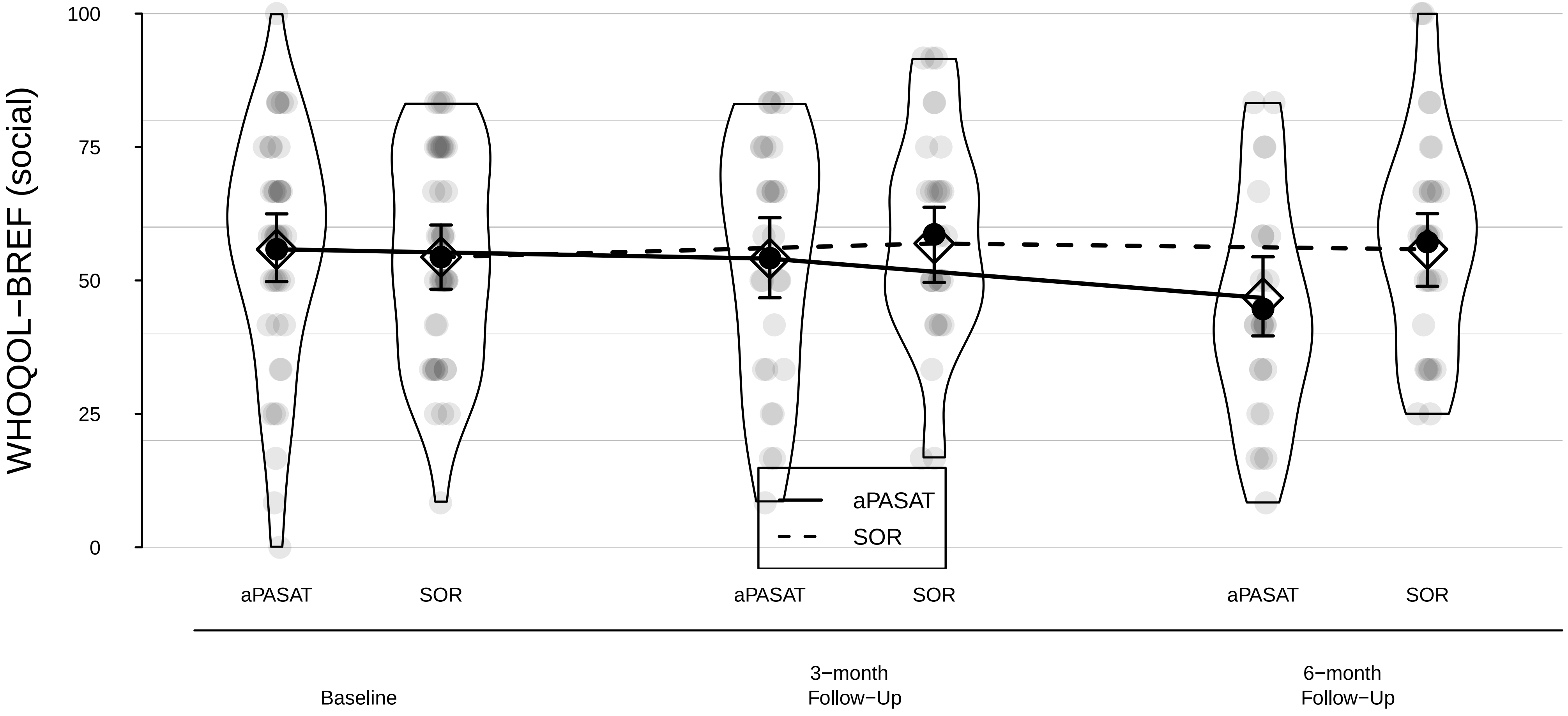

Note. WHOQOL−BREF (social) = World Health Organization Quality of Life Brief Form; Grey dots represent individual data points.
Solid black dots show empirical mean values. Diamond shapes represent model−based estimates values with 95% confidence intervals.
The width of the plot reflects the distribution of data. aPASAT = adaptive Paced Auditory Serial Addition Test Training; SOR = Speed of Response Training

Figure S16

Model−Based Change Trajectories in Quality of Life (environmental)

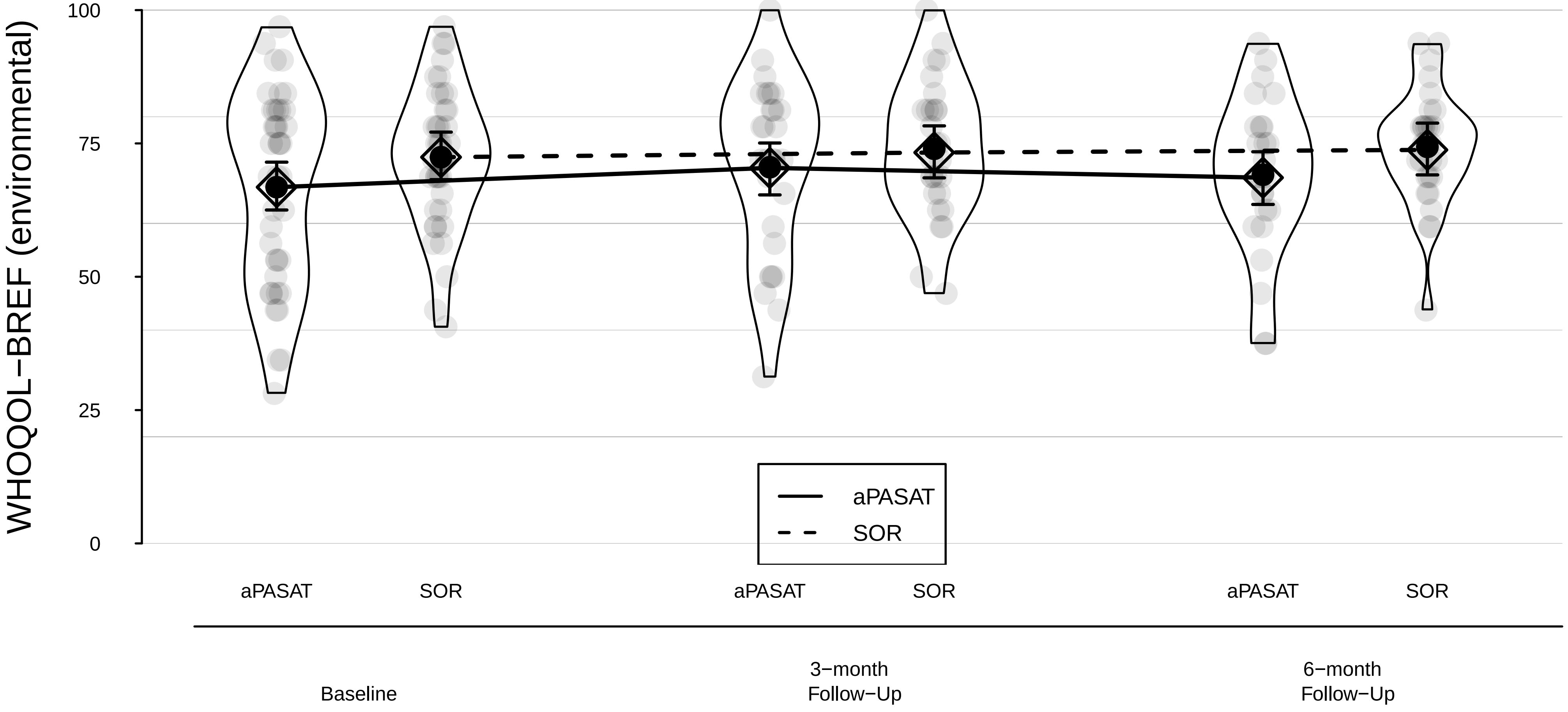

Note. WHOQOL−BREF (environmental) = World Health Organization Quality of Life Brief Form; Grey dots represent individual data points. Solid black dots show empirical mean values. Diamond shapes represent model−based estimates values with 95% confidence intervals. The width of the plot reflects the distribution of data. aPASAT = adaptive Paced Auditory Serial Addition Test Training; SOR = Speed of Response Training

Figure S17

Model−Based Change Trajectories in Work and Social Adjustment

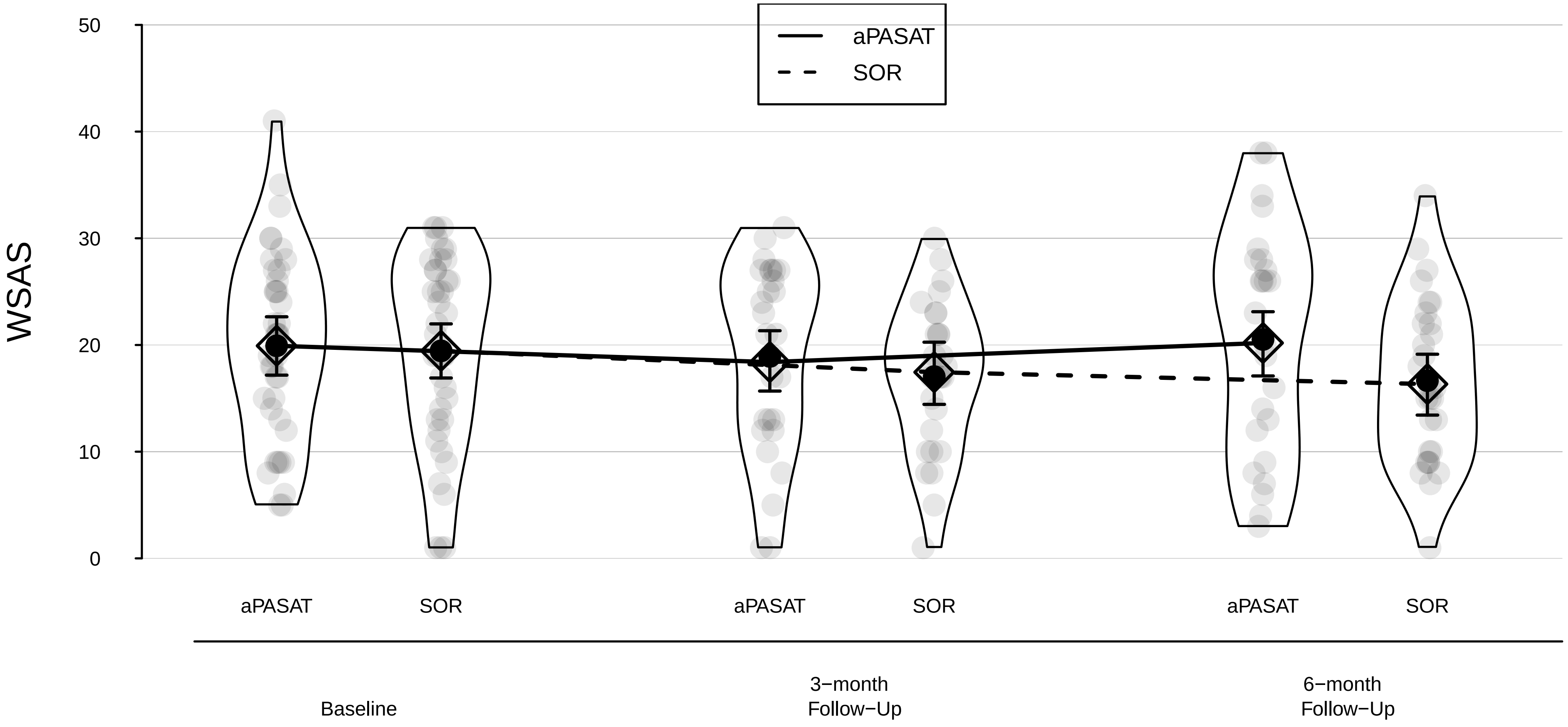

Note. WSAS = Work and Social Adjustment Scale; Grey dots represent individual data points.
Solid black dots show empirical mean values. Diamond shapes represent model−based estimates values with 95% confidence intervals.
The width of the plot reflects the distribution of data. aPASAT = adaptive Paced Auditory Serial Addition Test Training; SOR = Speed of Response Training

Figure S18

Model−Based Change Trajectories in Brooding

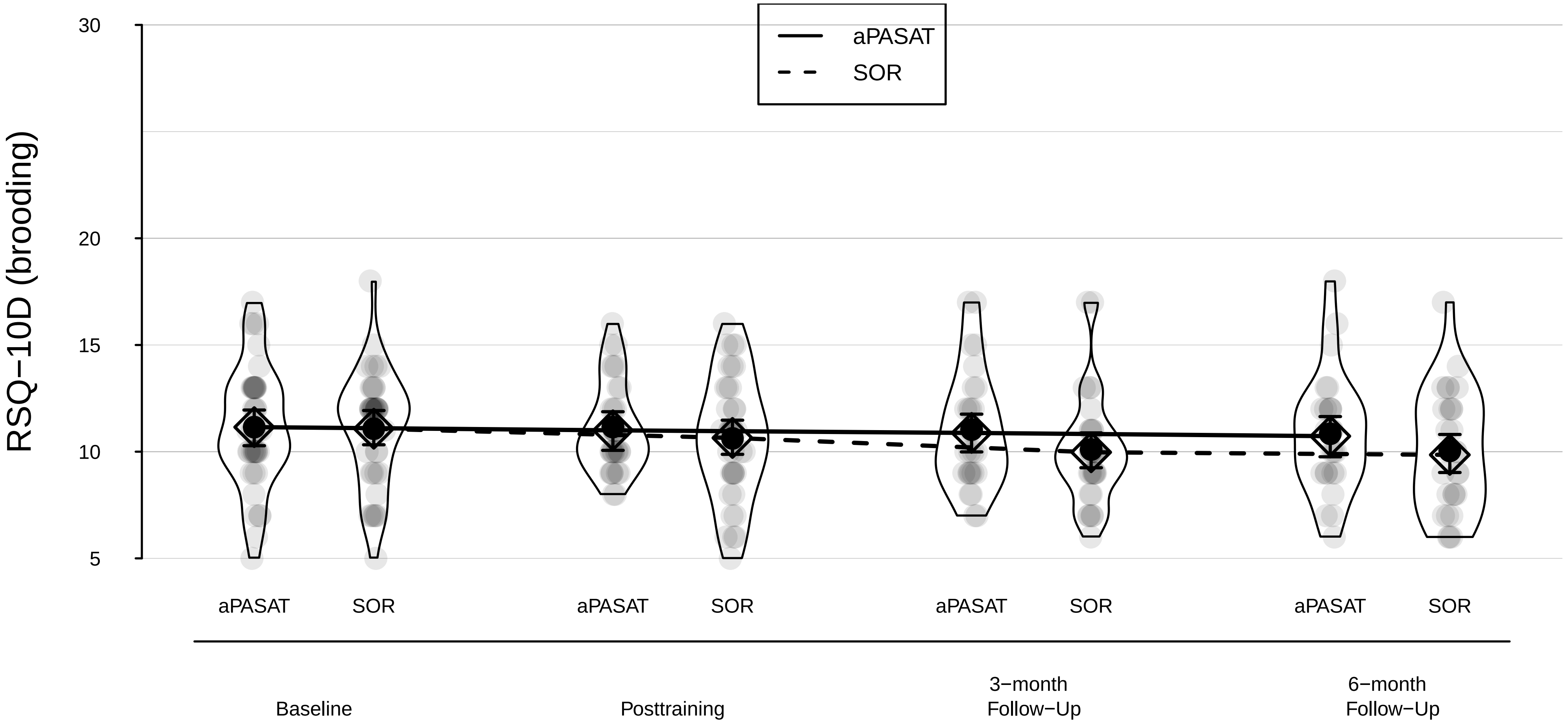

Note. RSQ−10D (brooding) = Response Style Questionnaire (brooding subfacet); Grey dots represent individual data points.
Solid black dots show empirical mean values. Diamond shapes represent model−based estimates values with 95% confidence intervals.
The width of the plot reflects the distribution of data. aPASAT = adaptive Paced Auditory Serial Addition Test Training; SOR = Speed of Response Training

Figure S19

Model−Based Change Trajectories in Rumination Reflection

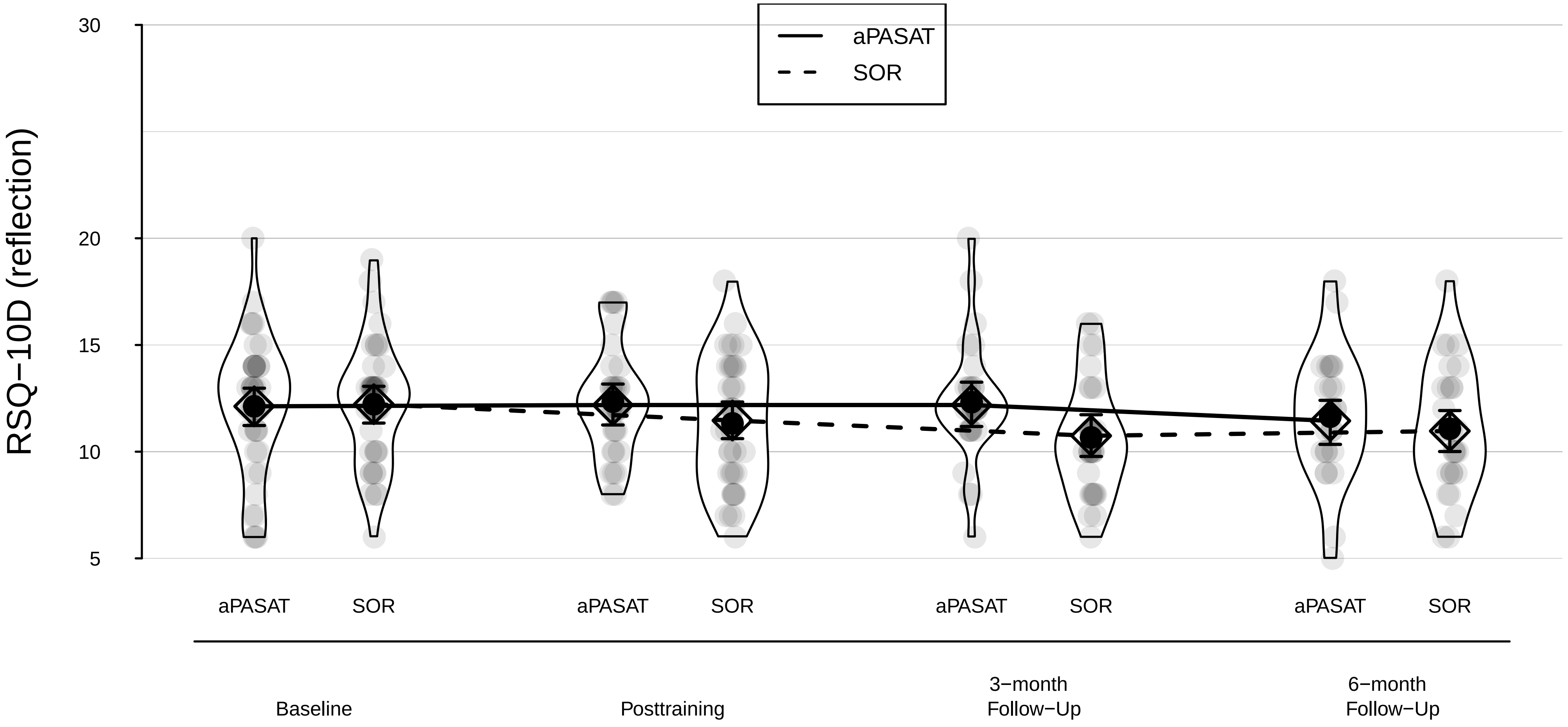

Note. RSQ−10D (reflection) = Response Style Questionnaire (reflection subfacet); Grey dots represent individual data points.
Solid black dots show empirical mean values. Diamond shapes represent model−based estimates values with 95% confidence intervals.
The width of the plot reflects the distribution of data. aPASAT = adaptive Paced Auditory Serial Addition Test Training; SOR = Speed of Response Training

Figure S20

Model−Based Change Trajectories in Global Mental Distress (Per Protocol Analysis)

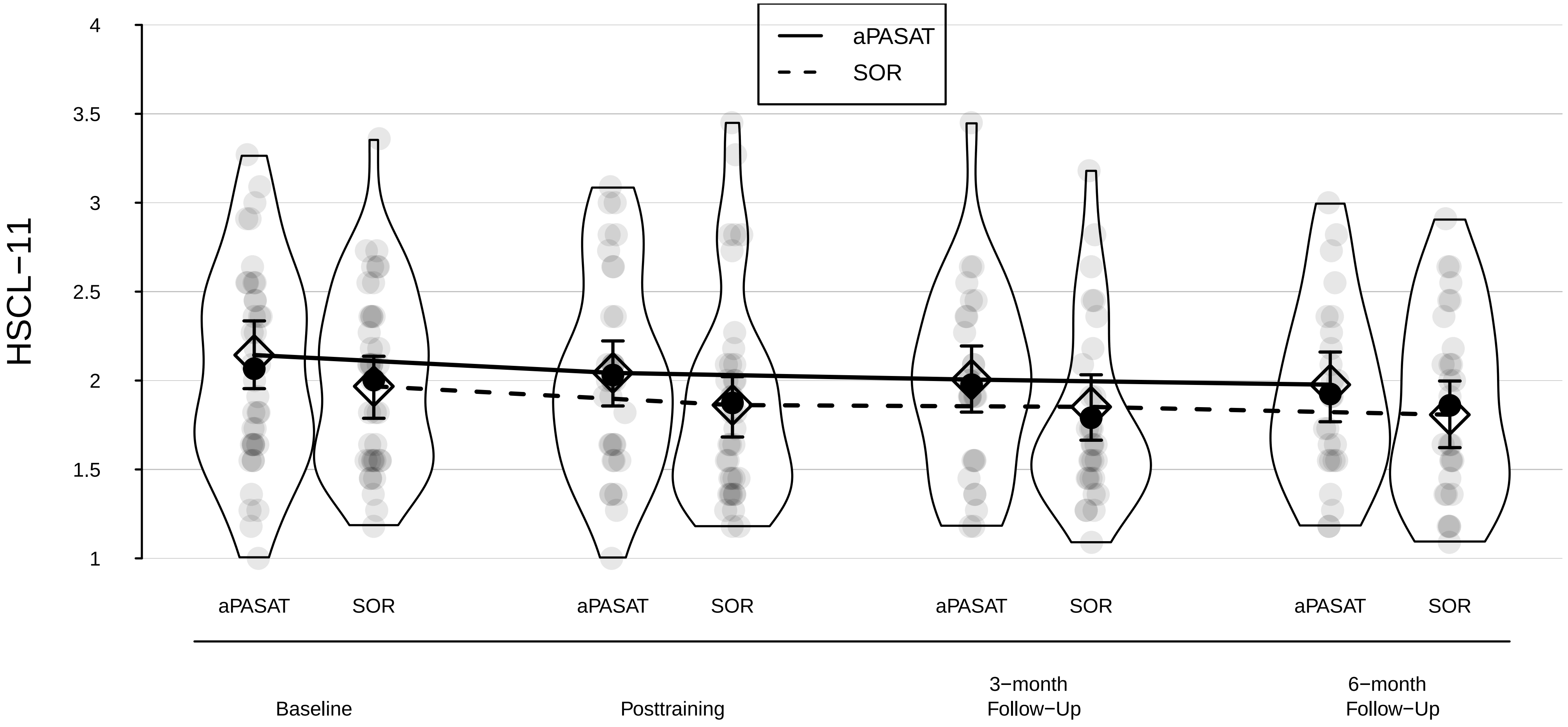

Note. HSCL−11 = Hopkins Symptom Checklist 11; Grey dots represent individual data points.
Solid black dots show empirical mean values. Diamond shapes represent model−based estimates values with 95% confidence intervals.
The width of the plot reflects the distribution of data. aPASAT = adaptive Paced Auditory Serial Addition Test Training; SOR = Speed of Response Training

Figure S21

Model−Based Change Trajectories in Rumination (Per Protocol Analysis)

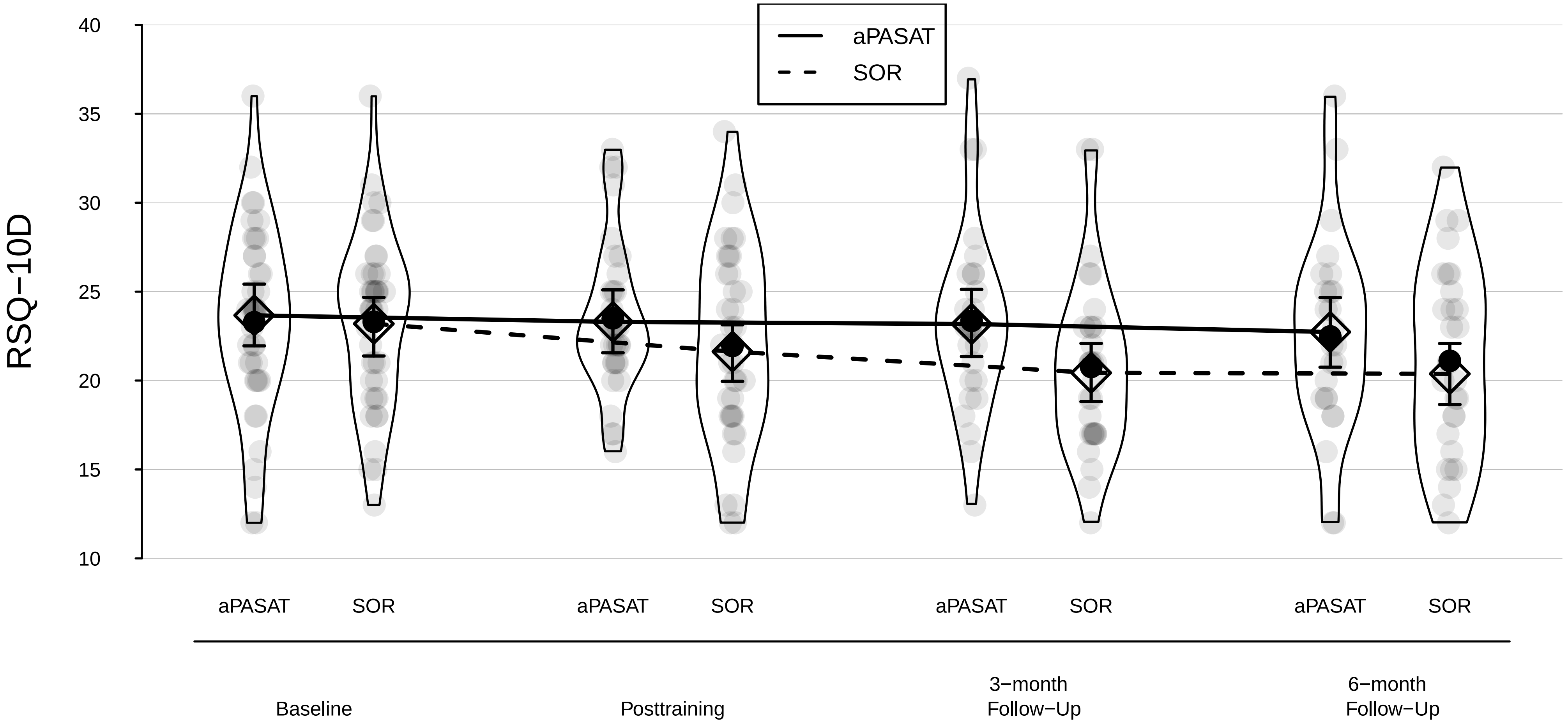

Note. RSQ−10D = Response Style Questionnaire (10−item version); Grey dots represent individual data points.
Solid black dots show empirical mean values. Diamond shapes represent model−based estimates values with 95% confidence intervals.
The width of the plot reflects the distribution of data. aPASAT = adaptive Paced Auditory Serial Addition Test Training; SOR = Speed of Response Training

Figure S22

Model−Based Change Trajectories in Repetitive Negative Thinking (Per Protocol Analysis)

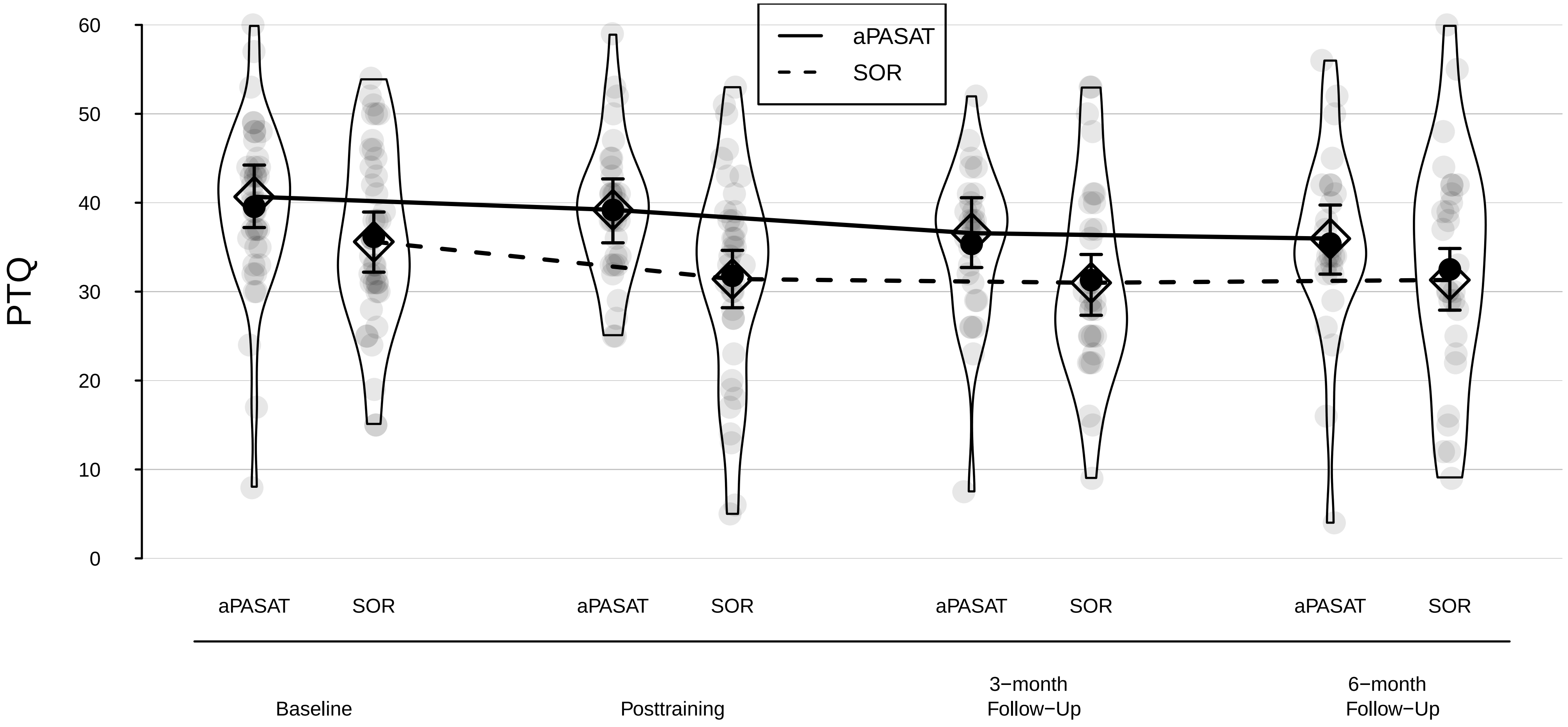

Note. PTQ = Perseverative Thinking Questionnaire; Grey dots represent individual data points.
Solid black dots show empirical mean values. Diamond shapes represent model−based estimates values with 95% confidence intervals.
The width of the plot reflects the distribution of data. aPASAT = adaptive Paced Auditory Serial Addition Test Training; SOR = Speed of Response Training

Figure S23

Model−Based Change Trajectories in Difficulties in Emotion Regulation (Per Protocol Analysis)

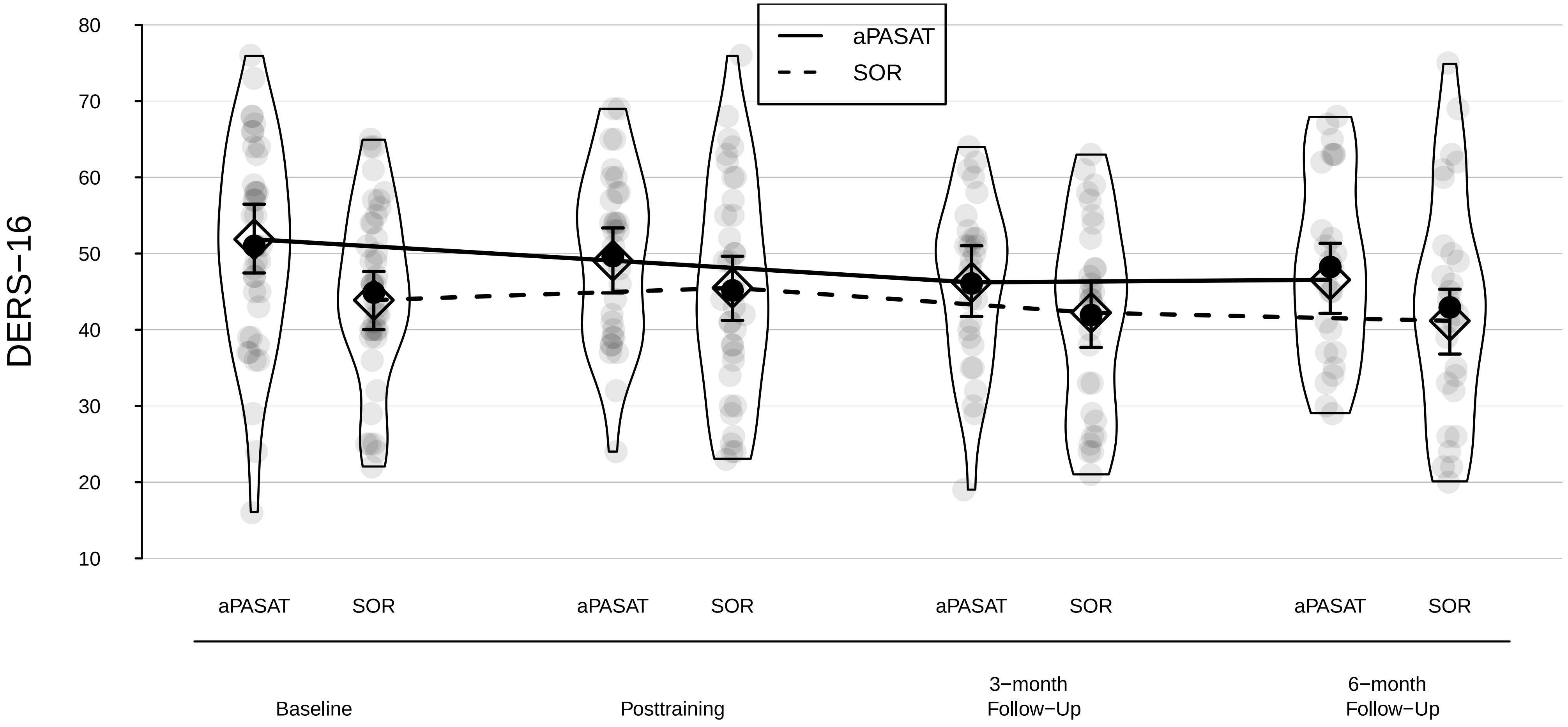

Note. DERS−16 = Difficulties in Emotion Regulation Questionnaire (16−item version); Grey dots represent individual data points.
Solid black dots show empirical mean values. Diamond shapes represent model−based estimates values with 95% confidence intervals.
The width of the plot reflects the distribution of data. aPASAT = adaptive Paced Auditory Serial Addition Test Training; SOR = Speed of Response Training

Figure S24

Model−Based Change Trajectories in Adaptive Cognitive Emotion Regulation (Per Protocol Analysis)

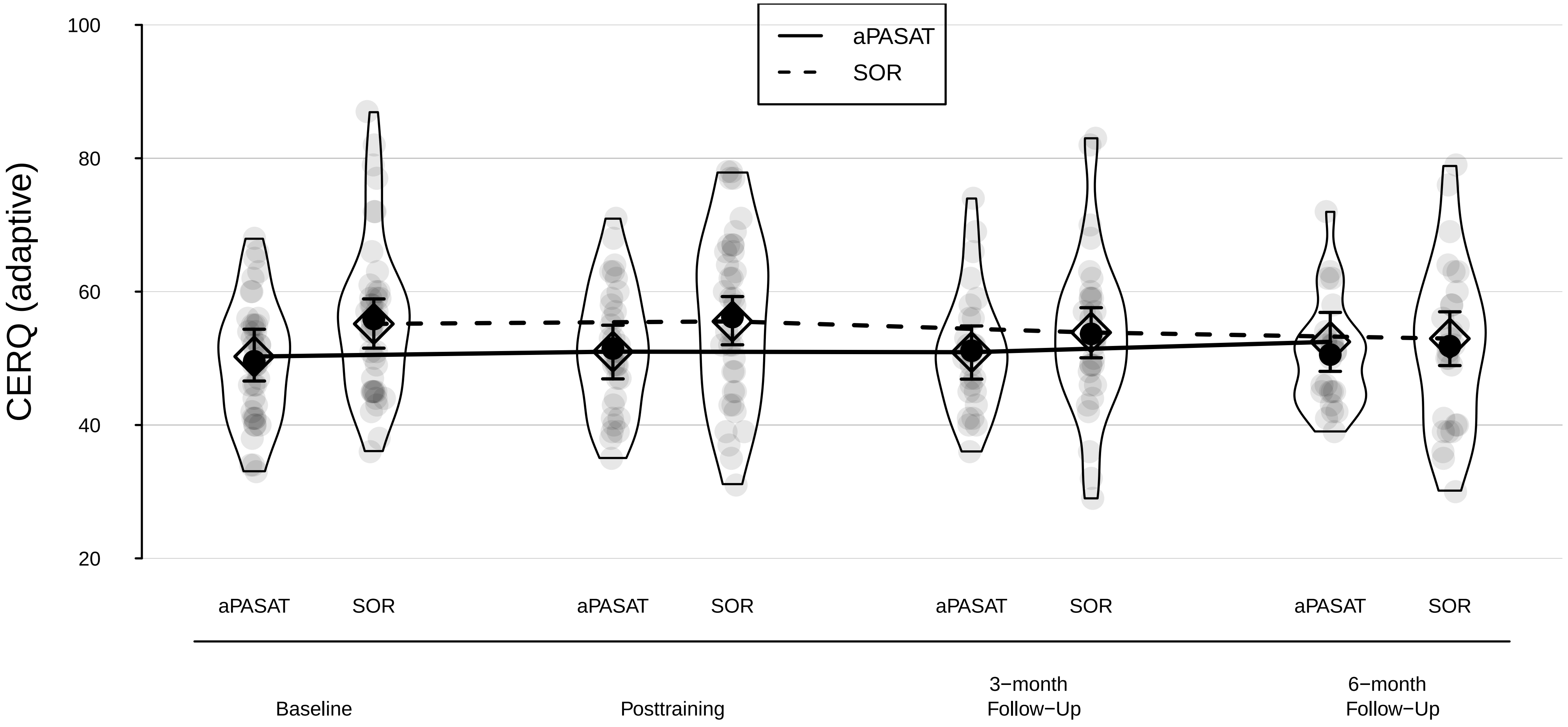

Note. CERQ (adaptive) = Cognitive Emotion Regulation Questionnaire (adaptive subscale); Grey dots represent individual data points. Solid black dots show empirical mean values. Diamond shapes represent model−based estimates values with 95% confidence intervals. The width of the plot reflects the distribution of data. aPASAT = adaptive Paced Auditory Serial Addition Test Training; SOR = Speed of Response Training

Figure S25

Model−Based Change Trajectories in Maladaptive Cognitive Emotion Regulation (Per Protocol Analysis)

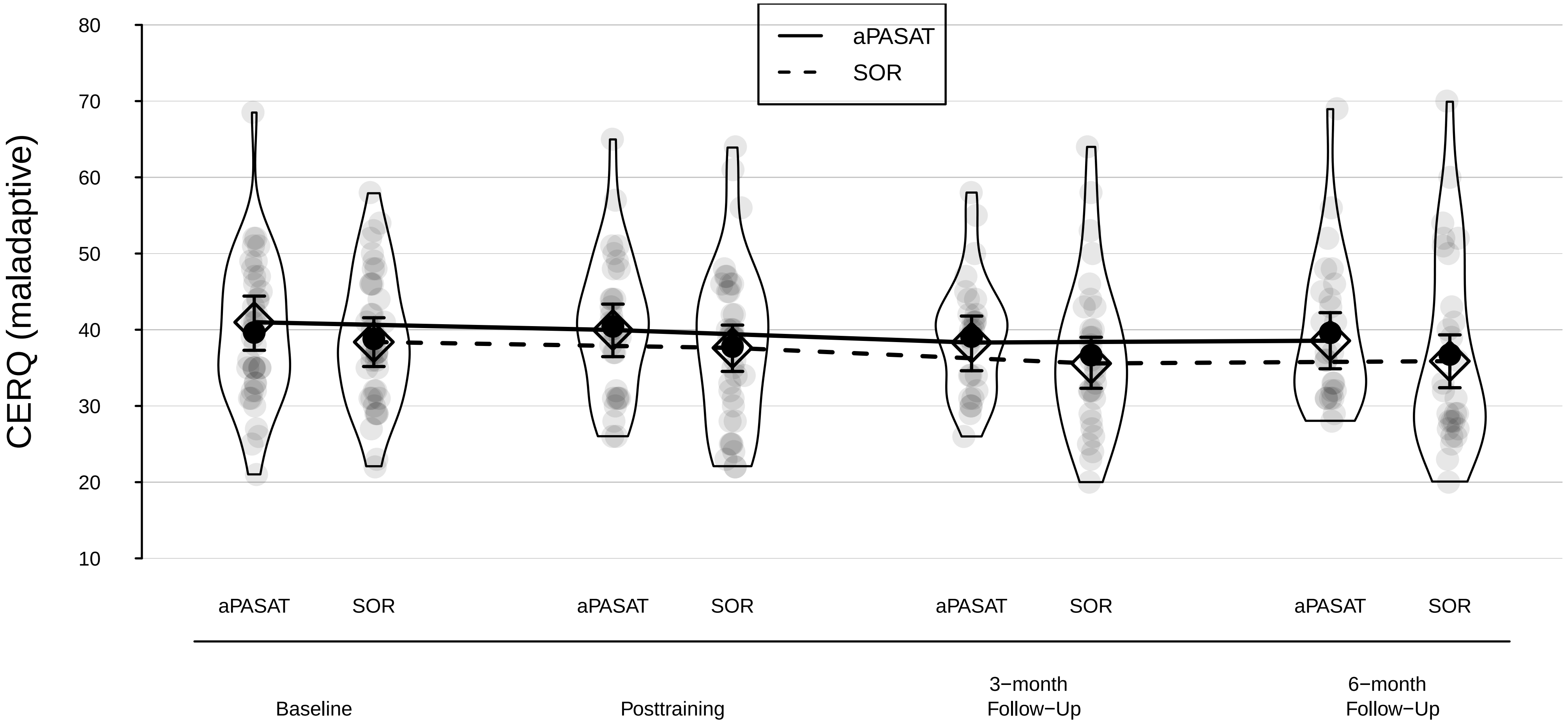

Note. CERQ (maladaptive) = Cognitive Emotion Regulation Questionnaire (maladaptive subscale); Grey dots represent individual data points. Solid black dots show empirical mean values. Diamond shapes represent model−based estimates values with 95% confidence intervals. The width of the plot reflects the distribution of data. aPASAT = adaptive Paced Auditory Serial Addition Test Training; SOR = Speed of Response Training

Figure S26

Model−Based Change Trajectories in Cognitive Control (Per Protocol Analysis)

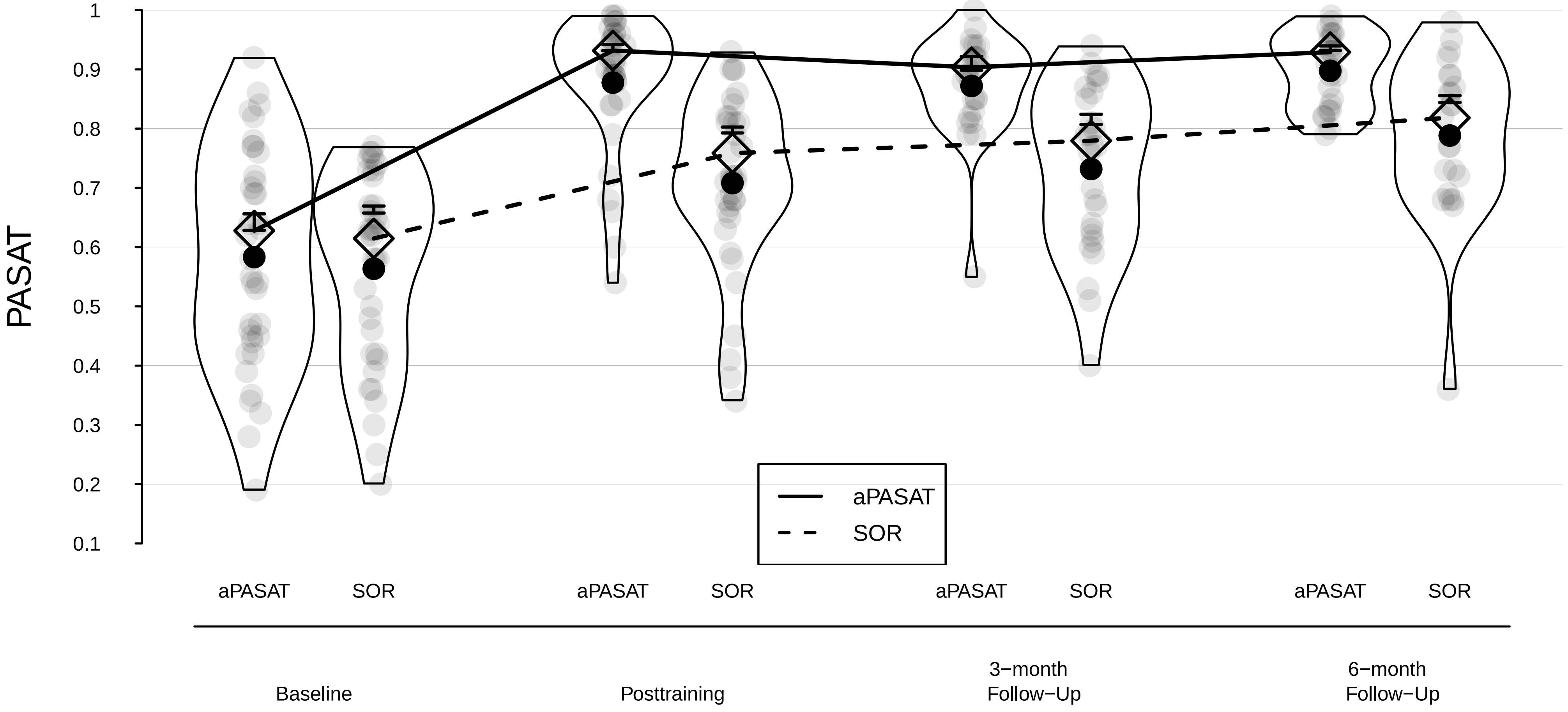

Note. PASAT = Paced Auditory Serial Addition Test; Grey dots represent individual data points.
Solid black dots show empirical mean values. Diamond shapes represent model−based estimates values with 95% confidence intervals.
The width of the plot reflects the distribution of data. aPASAT = adaptive Paced Auditory Serial Addition Test Training; SOR = Speed of Response Training
